# Supplementary material for: Typical and Aberrant Functional Brain Flexibility: Lifespan Development and Aberrant Organization in Traumatic Brain Injury and Dyslexia
Source: Brain Sci. 2019 Dec 16;9(12):380. doi: 10.3390/brainsci9120380 (PMC6956162; doi:10.3390/brainsci9120380)
Supplement: Supplementary file 1 [file brainsci-09-00380-s001.pdf]

Supplementary Material

Typical and aberrant functional brain flexibility: lifespan development and aberrant organization in traumatic brain injury and dyslexia

Stavros I. Dimitriadis[1-6*], Panagiotis G. Simos[7,8], Jack M. Fletcher[9], Andrew C. Papanicolaou[10,11]

[1]Division of Psychological Medicine and Clinical Neurosciences, School of Medicine, Cardiff University, Cardiff, United Kingdom

[2]Cardiff University Brain Research Imaging Centre, School of Psychology, Cardiff University, Cardiff, United Kingdom

[3]School of Psychology, Cardiff University, Cardiff, United Kingdom

[4]Neuroinformatics Group, Cardiff University Brain Research Imaging Centre, School of Psychology, Cardiff University, Cardiff, United Kingdom

[5]Neuroscience and Mental Health Research Institute, Cardiff University, Cardiff, United Kingdom

[6]MRC Centre for Neuropsychiatric Genetics and Genomics, School of Medicine, Cardiff University, Cardiff, United Kingdom

[7]School of Medicine, University of Crete, Greece

[8]Institute of Computer Science, Foundation for Research and Technology, Greece

[9] Department of Psychology, University of Houston, Houston, Texas, TX, USA

[10]Division of Clinical Neurosciences, Department of Pediatrics, University of Tennessee Health Science Center, Memphis, TN, USA

[11]Le Bonheur Neuroscience Institute, Le Bonheur Children's Hospital, Memphis, TN, USA

* Correspondence should be addressed to:
S.I. Dimitriadis, Institute of Psychological Medicine and Clinical Neurosciences, Cardiff University, School of Medicine, Cardiff, United Kingdom
email: DimitriadisS@cardiff.ac.uk , stidimitriadis@gmail.com, Tel: +44-02920-876506

1. Preprocessing

The MEG data underwent artifact reduction using Matlab (The MathWorks, Inc., Natick, MA, USA) and Fieldtrip routines (Oostenveld et al., 2011). Independent component analysis (ICA) was used to separate cerebral from non-cerebral activity using the extended Infomax algorithm as implemented in EEGLAB (Delorme and Makeig, 2004). The data were whitened and reduced in dimensionality using principal component analysis with a threshold set to 95% of the total variance (Delorme and Makeig, 2004, Escudero et al., 2011). Kurtosis, Rényi entropy, and skewness values of each independent component were used to identify and remove ocular and cardiac artifacts. A given component was considered artifactual if, after normalization to zero mean and unit variance, more than 20% of its values exceeded 2 SDs from the mean (Escudero et al., 2011, Dimitriadis et al., 2013a, Antonakakis et al., 2013, 2015). Axial gradiometer recordings were transformed to planar gradiometer field approximations using the *sincos* method implemented in Fieldtrip (Oostenveld et al., 2011).

2. Intra and Inter-Frequency Coupling Estimators and statistical filtering

The center of the sliding window moved forward at 0.1-sec steps and the set of, were re-estimated leading to a series of 900 functional connectivity graphs (^{TV}FCGs) each representing a snapshot of the spatial profile of each connectivity index during the recording session.
Surrogate data were generated by cutting at a single random time point of each time series and swapping the two resulting time courses (Dimitriadis et al. 2013). This approach was chosen over the shuffling-based surrogate analysis which significantly alters the spectrum of the time series and also impairs the non-stationarity of brain activity. Here we assessed determinism/stochastisity and linearity/nonlinearity of the surrogate time series using the Delay Vector Variance (DVV) method which relies on signal predictability in phase space to characterize the time series (Dimitriadis et al., 2017b). Preliminary analyses using DVV indicated that the surrogate data produced in this method displayed similar frequency composition and stationarity as compared to the recorded time-series.

2.1. *Amplitude Envelope Correlation (AEC)*

Surrogate data analyses ensured that significant observed AEC values could not have emerged from time series with zero amplitude envelope coupling. The final AEC data set consisted of the highest, significant correlations across all frequencies and frequency pairs (if more than two frequencies or pairs of frequencies were significant for a given pair of sensors we selected the one with the maximum AEC value). Finally, the significant, dominant AEC values for each pair of sensors and across sliding windows were integrated into a 4D graph (^{TV}AEC 4D array of size [900 x 248/275 x 248/275]). In this array significant AEC interactions were indicated by a value of 1, with zeros indicating non-significant AEC interactions.

2.2. Phase-to-amplitude Cross-Frequency Coupling (CFC, cross-frequency iPLV)

Let $x(i_{sensor}, t)$, be the MEG activity recorder at the i_{sensor}-th site, and t=1, 2,.... T the successive time points. Given two band-limited signals $x(i_{sensor},t)$ and $x(j_{sensor},t)$, cross-frequency coupling was estimated by allowing the phase of the lower frequency (LF) oscillation to modulate the amplitude of the higher frequency (HF) oscillation. The complex analytic representations of each signal $z_{LF}(t)$ and $z_{HF}(t)$ were derived via the Hilbert transform (HT[.]).

$$z_{LF}(t) = HT[x_{LF}(t)] = |z_{LF}(t)|\, e^{i\phi_{LF}(t)} = A_{LF}(t)\, e^{i\phi_{LF}(t)},\; z_{HF}(t) = HT[x_{HF}(t)] = |z_{HF}(t)|\, e^{i\phi_{HF}(t)} = A_{HF}(t)\, e^{i\phi_{HF}(t)}$$

Next, the envelope of the higher-frequency oscillation $A_{HF}(t)$ was bandpass-filtered within the range of the LF oscillation and the resulting signal was submitted to an additional Hilbert transform to derive its phase dynamics component φ'(t):

$$z'(t) = HT[\; A_{HF,LF}(t)\;] = |z'(t)|\, e^{i\phi'_{HF}(t)} = |z'(t)|\, e^{i\phi_{LF\to HF}(t)}$$

which expresses the modulation of the amplitude of the HF oscillation by the phase of the LF oscillation. Phase consistency between the two timeseries, corresponding to PAC strength, was measured by means of the imaginary portion of the Phase Locking Value (iPLV; Lachaux et al., 1999) defined as follows:

$$\boldsymbol{iPLV = \frac{1}{T} * \left| i\Big(\sum_{t=1}^{T} e^{i(\varphi_i(t)\; - \varphi_j(t))}\Big) \right|}$$

The imaginary portion of PLV is considered to be less susceptible to volume conduction in assessing CFC interactions and was used in all subsequent analyses. While iPLV is not affected by volume conduction, it may be sensitive to changes in the angle between two signals, which do not necessarily imply a PLV change. In general, iPLV is only sensitive to non-zero-phase lags and is thus resistant to instantaneous self-interactions associated with volume conduction (Nolte et al., 2004).

This procedure, the implementation details of which can be found elsewhere (Dimitriadis et al., 2010a), resulted in 900 time-varying PAC graphs per participant, each serving as an instantaneous snapshot of the surface network. There were 86, 82, 80, 77, 70, 60, and 40 (=455) possible pairs in the δ, θ, α1, α2, β1, β2, and γ1 bands, respectively. For instance, using 1Hz frequency bins there were 90-4 = 86 interacting pairs for δ phase.

For each pair of sensors, in each sliding window and for each subject, the maximum PAC score was determined for each combination of modulating frequency bands (fφ; from δ to γ) and modulated frequencies (*fa*; ranging from 1 to 90Hz in 1Hz steps) using the following equation:

$$(\,^{\wedge}f_{\varphi},\,^{\wedge}f_{\alpha}) = \underset{f_{\varphi}-f_{\alpha}}{argmax}\left(PAC(f_{\varphi}, f_{\alpha})\right) \quad (1)$$

In this manner we determined if the prominent CFC interaction (indexed by the maximum PAC value), and specifically the frequency of the low-frequency phase, was associated with the frequency of maximum power. If the two frequencies were identical this would imply that the observed prominent CFC interaction was driven by the power of the dominant frequency and not its phase. In order to ensure that we did not include in further analyses CFC interactions of this type, we required a minimum frequency difference of 1Hz distance between the two frequencies (frequency of the low-frequency phase, frequency of the higher power), the one identified by equation 1 (maximum PAC value) and the one associated with the maximum spectral power, within the range of high amplitude. Surrogate data analyses further ensured that a very low p value associated with a given observed PAC indicated that it could not have emerged from time series with zero cross-frequency phase-amplitude coupling. To be included in surrogate analyses a given PAC value would have to meet an additional criterion, namely that the frequency of the modulated HF should be distinct

from the frequency associated with maximum power. Here, we adopted a criterion of at least 1 Hz difference between the two frequencies.

The dominant PAC values for each pair of sensors and across sliding windows were integrated across frequency bins yielding 28 possible pairwise PAC estimates among the eight frequency bands. For each participant the resulting ^{TV}PAC profiles constituted two 4D arrays of size [28 (pairs of frequencies) x 900 (time windows) x 248/275 (sensors) x 248/275 (sensors)]. The first array contained the PAC values, and the second array the corresponding p-value. In a third array of size [2 x 900 x 248/275 x 248/275], dICMs were integer-coded (e.g., 1 for δ, 2 for θ,…., 11 for δ→α, 18 for θ→β1).

2.3. Intra-Frequency Phase-to-Phase Coupling (same-frequency iPLV)

Intra-frequency phase coupling was estimated using the Hilbert phase transform implemented in the following formula adapted from the following equation:

$$\boldsymbol{iPLV = \frac{1}{T} * \left| i(\sum_{t=1}^{T} e^{i(\varphi_i(t)\ -\varphi_j(t))}) \right|}$$

Based on surrogate data analyses an p value was assigned to each observed iPLV value, with very low p values suggesting that the observed iPLV could not have emerged from time series with zero phase coupling. Finally, the dominant iPLV mode for each sensor pair was determined based on the highest, statistically significant iPLV value. The significant, dominant iPLV values for each pair of sensors and across sliding windows were integrated into two 4D arrays (^{TV}iPLV) of size [8 x 900 x 248/275 x 248/275]). The first array contained the iPLV values and the second array the corresponding p-value. In a third array of size [2 x 900 x 248/275 x 248/275], the dICMs were integer-coded (e.g., 1 for δ, 2 for θ,…., 11 for δ→α, 18 for θ→β1).

2.4. Delay Symbolic Transfer Entropy (dSTE)

In principle, asymmetric dependences between coupled systems can be detected with measures of mutual information (Dimitriadis et al., 2010) taking into account the dynamics of information transfer. Transfer entropy (Shannon & Weaver, 1949), which is related to Granger causality (Granger, 1969), was introduced in order to distinguish the driving and responding elements within a network. Through proper conditioning of transition probabilities this quantity has been shown to be superior to the standard time-delayed mutual information, which fails to distinguish information, which is actually exchanged, from shared information due to common history and input signals. Various techniques have been proposed to estimate transfer entropy from observed data. Most techniques, however, make great demands on the data, require fine-tuning of parameters, and are highly sensitive to noise contributions, which limits the use of transfer entropy to field applications (Granger, 1969, Verdes, 2005).

The algorithmic steps employed to transcribe the temporal dynamics of each pair of sensors into two distinct symbolic timeseries that share a common codebook (set of symbols) are described below. The size and content of the codebook is data-dependent and estimated causal relationships are to be inferred from a different pair of recorded signals each time. The unsupervised nature of the algorithm reduces computational burden (Dimitriadis et al., 2016a,c,d). Given signals $^{A}x_t$ and $^{B}x_t$ recorded from sensors A and B, respectively, time-delay vectors are first reconstructed from each time series. These vectors take the form of $\mathbf{x_t} = \{x_t, x_{(t+\tau)}, \ldots, x_{(t+(m-1)\tau)}\}$, where m is the embedding dimension, τ denotes

the time lag and t=1,2,..,T runs over the time points. Then, the two individual sequences of time-delay vectors are collectively gathered in data matrices in the form of:

$$^{A}\mathbf{X}_{[T\times m]} = [^{\mathbf{A}}\mathbf{x}_1 | ^{A}\mathbf{x}_2 \mid \ldots \mid ^{\mathbf{A}}\mathbf{x}_T] \quad \& \quad ^{B}\mathbf{X}_{[T\times m]} = [^{B}\mathbf{x}_1 | ^{B}\mathbf{x}_2 \mid \ldots \mid ^{B}\mathbf{x}_T]$$

Next, the two trajectories are brought to a common reconstructed state space by forming the overall data matrix

$$^{AB}\mathbf{X}_{[2T\times m]} = [\,^{\mathbf{A}}\mathbf{X} \mid ^{B}\mathbf{X}\,] \quad (2)$$

Partitioning all the tabulated m-dimensional vectors into groups of homogenous patterns is the most direct way to summarize the temporal variations in the two time series and describe them with a common vocabulary. In our approach, a codebook of k code vectors is designed by applying the NG algorithm to the data matrix described by Eq. 2. The NG algorithm is an artificial neural network model, which converges efficiently to a small number $k << T$ of codebook vectors $\{\mathbf{M_i}\}_{i=1:k}$ using a stochastic gradient descent procedure with a soft-max adaptation rule that minimizes the average distortion error (Martinetz et al., 1993). In the encoding stage, each of the $2T$ vectors was assigned to the nearest code vector. By replacing the original vectors with the assigned code vectors, the two vectorial time series can be reconstructed with a measurable error. If we denote the reconstructed (i.e. decoded) version of the vectorial time series as $^{AB}\mathbf{x}^{\mathbf{rec}}(t)$, the fidelity of the overall encoding procedure can be estimated via the index described in the following equation, which reflects the total distortion error divided by the total dispersion of the original vectors:

$$n_{Distortion} = \frac{\sum_{t=1}^{2T} \left\| ^{AB}\mathbf{x}(t) - ^{AB}\mathbf{x}^{\mathbf{rec}}(t) \right\|^2}{\sum_{t=1}^{2T} \left\| ^{AB}\mathbf{x}(t) - \bar{x} \right\|^2}, \quad \bar{\mathbf{x}} = \frac{1}{2T} \sum_{t=1}^{2T} {^{AB}\mathbf{x}(t)}$$

The smaller the $n_{Distortion}$, the better the encoding. This index becomes smaller as k increases, and reaches a plateau at a relatively small value of k. In the present study, we considered encoding to be acceptable if it was produced by the smallest k and if $n_{Distortion}$ was less than 5%. The NG algorithm was repeatedly applied at progressively higher k values to determine the optimal k_0, which in turn identified the codebook to use in the subsequent symbolization scheme. At the vector-quantization stage, each $^{\mathbf{A}}\mathbf{X}$ and $^{\mathbf{B}}\mathbf{X}$ vector was assigned (according to the nearest-prototype rule) to the most similar among the derived code-vectors $\{\mathbf{M_i}\}_{i=1:ko}$. This step completed the mapping of the recorded time series to corresponding symbolic time series $^{A}s_t$ and $^{B}s_t$, $t = 1, 2, \ldots T$, which in mathematical notation reads as follows:

$$[^{A}x_t,\ ^{B}x_t] \in R^2$$
$$^{\mathbf{A}}\mathbf{x_t} \xrightarrow{VQ} \mathrm{M}_{j_1} \in \{M_i\}_{i=1}^{k_o}, M_i \in R^m \qquad ^{B}\mathbf{x_t} \xrightarrow{VQ} \mathrm{M}_{j_2} \in \{M_i\}_{i=1}^{k_o}, M_i \in R^m$$
$$^{A}x_t \rightarrow {^{A}s_t} = \mathrm{j}_1(\mathrm{t}) \;,\quad ^{B}x_t \rightarrow {^{B}s_t} = \mathrm{j}_2(\mathrm{t}) \;,\quad \mathrm{j}_1, \mathrm{j}_2 \in \{1,2,..,k_o\}$$

In the derived symbolic time series, the temporal dynamics of a pair of neural subsystems are encoded as transitions among adaptively-defined (i.e. data-dependent) symbols. We adopted the Ragwitz criterion for optimizing the embedding dimension d and the embedding delay τ. Optimality of the embedding dimension refers to minimal prediction error for future samples of the time series. The Ragwitz criterion predicts the future of a signal based on estimates of the probability densities of future values of its nearest neighbors after embedding. The adopted method was based on the minimization of mean squared prediction error (Ragwitz and Kantz, 2002; Lindner et al., 2011).

dSTE as an index of Effective Connectivity. Given a pair of symbolic sequences ${}^{A}s_t$ and ${}^{B}s_t$, the relative frequency of occurrence of symbols can be used to estimate joint and conditional probabilities, and to define symbolic transfer entropy (STE) as follows

$$STE_{BA} = \sum p({}^{A}s_{t+\delta}, {}^{A}s_t, {}^{B}s_t) \log \frac{p({}^{A}s_{t+\delta} / {}^{A}s_t, {}^{B}s_t)}{p({}^{A}s_{t+\delta} / {}^{A}s_t)} \quad (3)$$

where the sum runs over all symbols and δ denotes a time step.

Effective connectivity is defined as “the influence one system exerts over another” (Granger, 1969; Ito et al., 2011). In the context of brain networks, effective interactions are directed from one sensor location to another. To account for the time-delay between neuromagnetic signals recorded from different sensors, Eq. 3 was adapted as follows:

$$dSTE_{BA} = STE_{BA}(d) = \sum p({}^{A}s_{t+1}, {}^{A}s_t, {}^{B}s_{t+1-d}) \log \frac{p({}^{A}s_{t+1} / {}^{A}s_t, {}^{B}s_{t+1-d})}{p({}^{A}s_{t+1} / {}^{A}s_t)}$$

where d is the time delay between the driving and the driven time series.

A log of base 2 is used so that STE_{BA} is expressed in bits. STE_{AB} is defined in complete analogy. The directionality index $\Delta dSTE_{AB}=dSTE_{AB}-dSTE_{BA}$ quantifies the preferred direction of information flow taking up positive values for a unidirectional coupling, with time series A as the driver, and negative values for time series B driving A. For symmetric bidirectional couplings ΔdSTE approximates zero. The formulation of transfer entropy with a time delay has been validated in a recent study, which presented a robust method for neuronal interaction delays (Wibral et al., 2013).

The significance of a particular type of interaction between two sensors was assessed through surrogate data (1000 time series of sensor B created through permutation of the elements of the symbolic time series ${}^{B}s_t$). A one-sided p-value was computed that corresponded to the percentage of surrogate $dSTE_{BA}$ values that were higher than the observed $dSTE_{BA}$. The final dSTE data set contained the strength, direction and delay of the significant and dominant pair of frequencies for each sensor. If more than two dSTE frequencies or frequency pairs were significant, the one with the maximum dSTE value was selected. For each participant the resulting ^{TV}dSTE profiles constituted two 4D arrays of size [28 (pairs of frequencies) x 900 (time windows) x 248/275 (sensors) x 248/275 (sensors)]. The first array contained the dSTE values and the second array the corresponding p-value. In a third array of size [2 x 900 x 248/275 x 248/275], the dominant dSTE interactions were integer-coded (e.g., 1 for δ, 2 for θ,…., 11 for δ→α, 18 for θ→β1).

2.5. Directed Phase Lag Index (dPLI)

The dPLI quantifies the phase difference between two time series A and B which can be classified as follows:

a) Signal A is consistently leading signal B in the phase domain (dPLI > 0.5), if the majority of phase differences fall within $0 <= \Phi(t) < \pi$

b) Inconclusive causality between A and B in either direction, if the phase difference of the two signals averages π radians

c) Signal B is consistently leading signal A in the phase domain (dPLI < 0.5), if the majority of phase differences fall within $-\pi <= \Phi(t) < 0$.

Significant, causal phase relationships for every pair of sensors were determined via surrogate data analyses to ensure that a very low p value indicated that the observed dPLI could not have emerged from time series lacking directed phase interactions. The highest, significant dPLI values for each pair of sensors, frequencies and sliding windows were stored in two ^{TV}dPLI 4D array of size [36 (within and between frequencies) x 900 (time windows) x 248/275 (sensors) x 248/275 (sensors)]. The first array contained the dPLI values and the second array the corresponding p-value. In a third array of size [2 x 900 x 248/275 x 248/275] the identity of dominant dPLI interactions was indicated by a numeric (integer) code.

3. Topological Filtering based on Orthogonal Minimal Spanning Tress (OMSTs)

The general concept of the OMST method can be described as follows: The initial (1st) MST connects all the V sensors through V-1 edges. Then, the V-1 connections of the 1st MST were substituted with zeros and a 2nd MST was estimated that connected all of the V sensors with minimal total distance, satisfying the constraint that it was orthogonal—i.e. it shared no common edges—with the 1st MST. Next, the V-1 connections of the 2nd MST were substituted with zeros and a 3rd MST was estimated that connected the sensors with the minimal total weight, subject to the constraint that it was orthogonal to the previous two constructed MST's (1st and 2nd). In general, an m-MST is orthogonal to all the previous (m-1) MST's, having exactly m(N-1) edges. The OMST method was applied as follows:

For each added N-1 edges correspond to a single OMST across multiple round OMTS, the objective function of Global Cost Efficiency (GCE) = GE-Cost was estimated, where Cost denotes the ratio of the total weight of the selected edges, over multiple iterations of OMST, divided by the total strength of the original full-weighted graph. The values of this formula range within the limits of an economical small-world network for healthy control participants. The network which is considered as functionally optimal is the one associated with the maximum value of the following quality formula:

$$J_{GCE}^{OMSTs} = GE - Cost$$

Topological filtering was applied next in order to reduce the spatial density of the sensor network featuring dICM weights. The curve maximum in the following Figure illustrates the optimization of the OMST algorithm for a healthy participant from the first temporal segment.

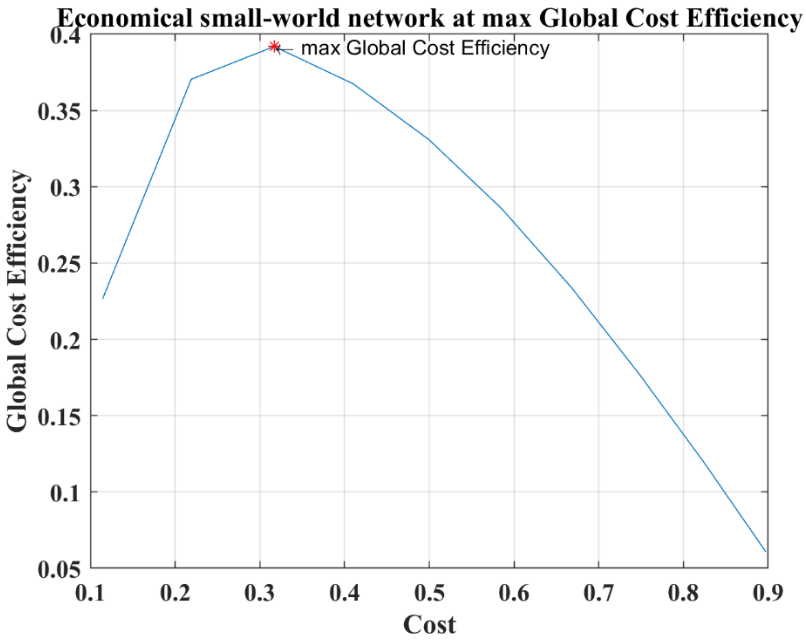

4. Supplementary metrics of brain activity & connectivity

For comparison purposes two well-established measures of brain activity and sensor interdependence were also computed, namely relative power spectrum and coherence. In contrast to dICM indices they provide static representations of the strength of oscillatory activity and within-frequency phase coupling, respectively.

4.1 Power Spectrum Analysis (RP)

Relative power was calculated to assess the relative contribution of each of the eight oscillatory components (0.5-4, 4-8, 8-10, 10-13, 13-15, 15-19, 20-29, and 30-45Hz corresponding to δ, θ, α_1, α_2, β_1, β_2, β_3, and γ bands) to global MEG signal power (Leuchter et al., 1993; Rodriguez et al., 1999). The MEG signals were converted to the frequency domain using the Welch periodogram in MATLAB R2012a (window length = 128 samples; window overlap = 64 samples; nfft = 128; sample frequency = 1024 Hz).

4.2 Imaginary Coherence between sensors (ImCOH)

Coherence is a widely used measure of linear dependence and phase consistency between two time series. Let $s_i(f)$ and $s_j(f)$ represent the complex Fourier transforms of two time series $x_i(t)$ and $x_j(t)$. For each of N epochs of the segmented time series, the cross-spectral density function $S_{ij}(f)$ of i and j is defined as:

$$S_{ijn} = s_{in}(f)s_{jn}^{*}(f)$$

and the single epoch power spectrum (auto-spectral density) as:

$$S_{in} = s_{in}(f)s_{in}^{*}(f)$$

where * indicates the complex conjugate. The coherency across *n* epochs is defined as the cross-spectrum normalized by the auto spectral density functions $x_i(t)$ and $x_j(t)$:

$$\hat{C}_{ij}(f) = \frac{|s_{ij}(f)|}{(S_i(f)S_j(f))^{1/2}}$$

Coherence is defined as the absolute value of coherency, i.e. the normalized amplitude of the complex cross-spectrum value:

$$Coh_{ij}(f) = |C_{ij}(f)|$$

In the present study we adopted the imaginary portion of coherence (Nolte et al., 2004) corresponding to the perpendicular presented to the real axis values of a complex number, positively increasing in magnitude to the left. In this manner, when two time series are completely simultaneous, i.e. they have zero-lag oscillatory activity, the value of ImCoh is zero:

$$\boldsymbol{Im\,C\,oh_{i,j}^{f}} = \mathfrak{R}[\boldsymbol{S_{i,j}(f)}] + \boldsymbol{i}\mathfrak{I}[\boldsymbol{S_{i,j}(f)}]$$

Thus ImCoh reflects only non-zero time-lag correlations which are presumably caused by interactions between distinct neuronal populations rather than common interference sources.

4.3 Multiscale Entropy (MSE)

Multiscale entropy (Costa et al., 2002, 2005) is a novel method to estimate the complexity of a finite length time series. For the purposes of the present study an MSE algorithm was developed for neuromagnetic time series using sample entropy (Richman and Moorman, 2000). Sample Entropy reflects the probability that sequences that match each other on the first two data points will also match on the next point (Costa et al., 2002). Assuming a time-series of length $N = \{x_1, x_2, x_3, ..., x_N\}$ with a constant time interval τ, we define a template vector of length m, such that $X_m(i) = \{x_i, x_{i+1}, x_{i+2}, ..., x_{i+m-1}\}$ and the Chebyshev distance function $d[X_m(i), X_m(j)]$ (i≠j). The number of vector pairs in template vectors of length m and m+1 is then counted having $d[X_m(i), X_m(j)] < r$ and denote it by B and A, respectively. Sample entropy is defined as follows:

$$SampEn = -\,log\frac{A}{B}$$

where

A = number of template vector pairs having $d[X_{m+1}(i), X_{m+1}(j)] < r$ of length m+1

B = number of template vector pairs having $d[X_m(i), X_m(j)] < r$ of length m

In contrast to traditional entropy indices, MSE provides a more direct and accurate measure of complexity by overcoming key limitations of the former, such as their susceptibility to bias introduced by surrogate data comparisons. The MSE method involves two steps:

1. A coarse-grained procedure is first applied to the time series. For a given time series, multiple coarse-grained time series are constructed by averaging the data points within non-overlapping windows of increasing length τ (see Figure S1). Each element of the coarse-grained time series, $y_j^{(\tau)}$, is calculated according to the equation:

$$y_j^{(\varsigma)} = \frac{1}{\tau} \sum_{i=(j-1)\tau+1}^{j\tau} x_i$$

where τ represents the scale factor and $1 \leq j \leq N/\tau$. The length of each coarse-grained time series is N/τ. For scale value = 1, the coarse-grained time series simply corresponds to the original time series.
2. Sample Entropy is then plotted as a function of scale values. As a “regularity statistic” SampEn searches for patterns within a time series and quantifies its degree of predictability or regularity (see Figure S2). Here, we estimated MSE separately for each participant, frequency band, and lobar section of the sensor array.

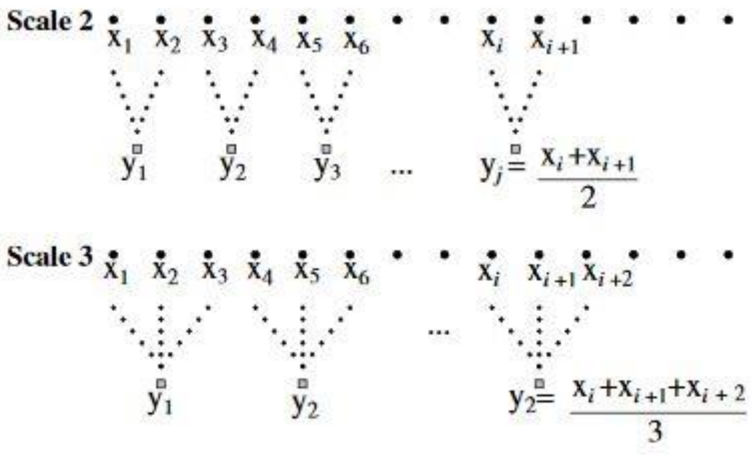

Figure S1: Schematic illustration of the coarse-grained procedure for scales 2 and 3. Adapted from Costa et al., 2002.

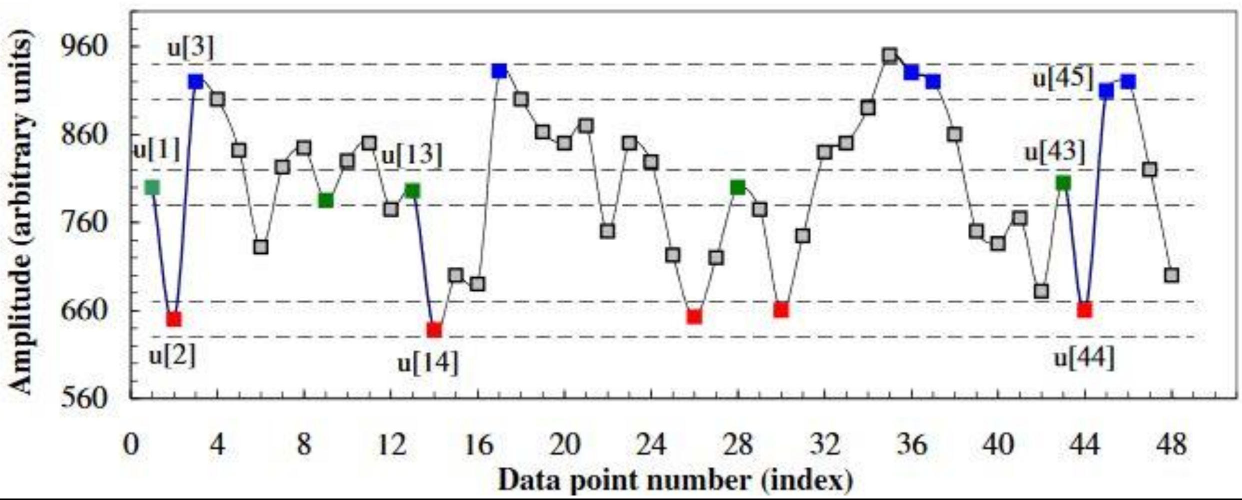

Figure S2: A simulated time series u[1], . . . , u[n] is shown to illustrate the procedure for calculating Sample Entropy for pattern length, m, of size 2, adopting a similarity criterion of r = 20 (r is a positive real number that is typically chosen to be between 10% and 20% of the sample deviation of the time series). Dotted horizontal lines around data points u[1], u[2] and u[3] represent u[1] ± r , u[2] ± r , and u[3] ± r , respectively. Two data values are considered indistinguishable if their absolute difference is ≤ r. Green data points match data point u[1]. Similarly, all red and blue points match data points u[2] and u[3], respectively. Consider the 2- component green-red template sequence (u[1], u[2]) and the 3-component green-red-blue (u[1], u[2], u[3]) template sequence. For the segment shown, there are two green-red sequences, (u[13], u[14]) and (u[43], u[44]), that match the template sequence (u[1], u[2]) but only one green-red-blue sequence that matches the template sequence (u[1], u[2], u[3]). Therefore, in this case, the number of sequences matching the 2-component template sequences is 2 and the number of sequences matching the 3-component template sequence is 1. These calculations were repeated for the next 2-component and 3-component template sequence: (u[2], u[3]) and (u[2], u[3], u[4]), respectively. The numbers of sequences that matched each of the 2- and 3-component template sequences were again counted and added to the previous values. This procedure was repeated for all other possible template sequences, (u[3], u[4], u[5]), . . . , (u[N-2], u[N-1], u[N]), to determine the ratio between the total number of 2-component template matches and the total number of 3-component template matches. Sample Entropy is the natural logarithm of this ratio and reflects the probability that sequences that match each other for the first two data points will also match for the next point (Costa et al., 2002).

5. Modelling Participant Age through Individual FI values

5.1. Prediction of participant age using Support Vector Regressors (SVR)

Drucker et al. (1997) extended the Support Vector Machine method to SVR in order to make continuous real-valued predictions. SVR retains some of the main features of SVM classification, although in the former method misclassified cases are penalized, whereas in SVR a penalty is served only for cases associated with extreme distances from the regression line in high-dimensional space (Dosenbach et al., 2010). Epsilon-insensitive SVR defines a tube of width ε, which is user defined, around the regression line in high-dimensional space. Any points within this tube carry no loss. In essence, SVR performs linear regression in high-dimensional space using epsilon-insensitive loss. The C parameter in SVR controls the trade-off between how strongly points beyond the epsilon-insensitive tube are penalized and the flatness of the regression line (larger values of C allow the regression line to be less flat; Dosenbach et al., 2010). SVR predictions described in the present analyses used epsilon-insensitive SVRs carried out in the Spider Machine Learning environment (Weston et al., 2005), as well as custom scripts run in MATLAB (R2010a; MathWorks, Natick, MA, USA). The parameters C and ε were tuned using a holdout subset of the respective dataset. An important feature of SVR is that it allows determination of the most important model features in predicting participant age, according to their corresponding weights.

5.2. *Prediction of participant age group using Multi-class Support Vector Machines (MC-SVM)*

Multi-class Support Vector Machines represent an extension of Support Vector Machines which are appropriate for binary classification (k = 2). Multi-Class pattern recognition (k>2 classes) are usually solved using a voting scheme based on combining many binary classification functions simultaneously. Here, we used a linear MC-SVM which involved training on all six age groups simultaneously, in order to build a unique decision function for each group (Crammer and Singer, 2001 and implemented in (http://www.cs.cornell.edu/people/tj/svm_light/svm_multiclass.html). Briefly, this method constructs $\frac{k(k-1)}{2}$ hyperplanes (1-against-1), each separating a given class from every other and applying a voting scheme for classification. The expected error probability in the test set was bounded by the ratio of the expected number of support vectors to the number of vectors in the training set:

$$E[P(\text{error})] = \frac{E[\text{number of support vectors}]}{(\text{number of training vectors}) - 1}$$

5.3. Cross Validation of SVRs and MC-SVMs

Cross validation of the age prediction and classification, respectively, was performed using three complementary schemes with each tool. In the first two, data from one MEG system were used to train the SVR and data from the second MEG system served as the testing set. The third scheme was applied to data aggregated across both MEG datasets. The prediction/classification capacity of each scheme was assessed using two alternative procedures: 5-fold cross-validation and leave-one-out-cross-validation (LOOCV; a method associated with the most unbiased test error estimates; Hastie et al., 2001). On each LOOCV round, one case is left out and the remaining cases are used as the training set. During each SVR round (LOOCV) or fold, the correlation between each feature and the dependent variable (age) was computed, and the features that had the highest absolute correlation values were selected for inclusion in the final prediction model.

6. Results

6.1. Identifying age-related neuromagnetic features

S.Table 1 presents results of various cross-validation schemes in the context of Support Vector Regression in predicting participant age based on the combination of 22 optimal features characterizing the short-term persistence of dICMs in 12 subnetworks (as defined by Mean Subgraph Strength) and 10 subnetworks (as defined by Fractional Occupancy). S.Table 1 illustrates the 22 optimal features that classify correct each participant to each age group based on Mean Subgraph Strength (MSS) and Fractional occupancy (FO). This model was able to predict participant age with a reasonable degree of accuracy according to the following regression equation: *Predicted Age* = 1.01 x *Actual Age* - 0.78 (R^2=0.893, $p < 2\times 10^{-9}$; Fig. S3). Corresponding results using Multi-Class Support Vector Machines to predict the correct age group of each participant are shown in S.Table 2. The most robust age prediction was achieved using the 5-fold cross validation method with data aggregated across both systems (as indicated by the capacity of the Support Regression model to account for 90% of individual variability in participant age and by 89% correct classification of participants to the appropriate age

group). Moreover, the comparability among the two data sets (Magnes-248, CTF-275) is evident by the high rates of successful age and age-group prediction when the Support Vectors were trained with the same set of dICM features from one system and tested with corresponding features from the other system.

S.Table 1. Support Vector Regressor results in predicting participant chronological age.

Data set		Features			
Training	Testing	dICM		RP, ImCOH, and MST	
		LOOCV	5-fold	LOOCV	5-fold
CTF-275	Magnes-248	R^2=0.865	R^2=0.876	R^2=0.789	R^2=0.779
		$p < 7.1\times 10^{-6}$	$p < 1.8\times 10^{-7}$	$p < 2.4\times 10^{-6}$	$p < 1.8\times 10^{-5}$
Magnes-248	CTF-275	R^2=0.834	R^2=0.869	R^2=0.812	R^2=0.801
		$p < 2.4\times 10^{-5}$	$p < 3.5\times 10^{-7}$	$p < 1.8\times 10^{-7}$	$p < 6.4\times 10^{-6}$
Mixed	Mixed	R^2=0.887	R^2=0.893	R^2=0.792	R^2=0.812
		$p < 5.1\times 10^{-8}$	$p < 2\times 10^{-9}$	$p < 7.8\times 10^{-6}$	$p < 2.2\times 10^{-7}$

S.Table 2. Percent classification of participants to the correct age group using Multi-Class Support Vector Machines.

Data set		Features			
Training	Testing	dICM		RP, ImCOH, and MST	
		LOOCV (%)	5-fold (%)	LOOCV (%)	5-fold (%)
CTF-275	Magnes-248	87.34±6.17	86.09±6.03	65.39±5.67	69.12 ± 5.45
Magnes-248	CTF-275	85.56±5.13	86.65±5.77	67.19±6.17	68.54±6.78
Mixed	Mixed	87.77±46.15	89.12±5.45	67.18±6.89	69.05±7.15

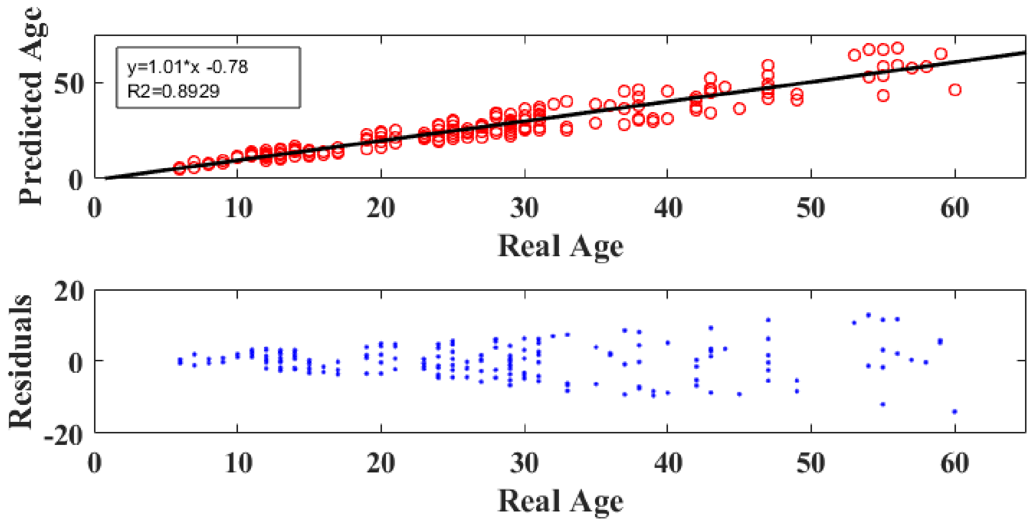

Figure S3. (Upper panel) Least squares regression of predicted over actual participant age in years based on Mean Subgraph Strength and Fractional Occupancy in 12 and 10 lobar subnetworks, respectively (listed in S.Table 2). (Lower panel) The distribution of model regression residuals as a function of participant age.

6.2. Age-Related Differences based on Dominant Intrinsic Coupling Modes (dICMs)

The spatial layout of dICMs which contributed significantly to the prediction of participant age is shown in the upper panels of Figures 1 and S4-14. The relative contribution of each of the 22 dICM features in the significant classification of participants to their correct age group through SVR is shown in the upper panel of S16. The prediction accuracy of participant age for each of the 22 features is displayed in the lower panel of S16.

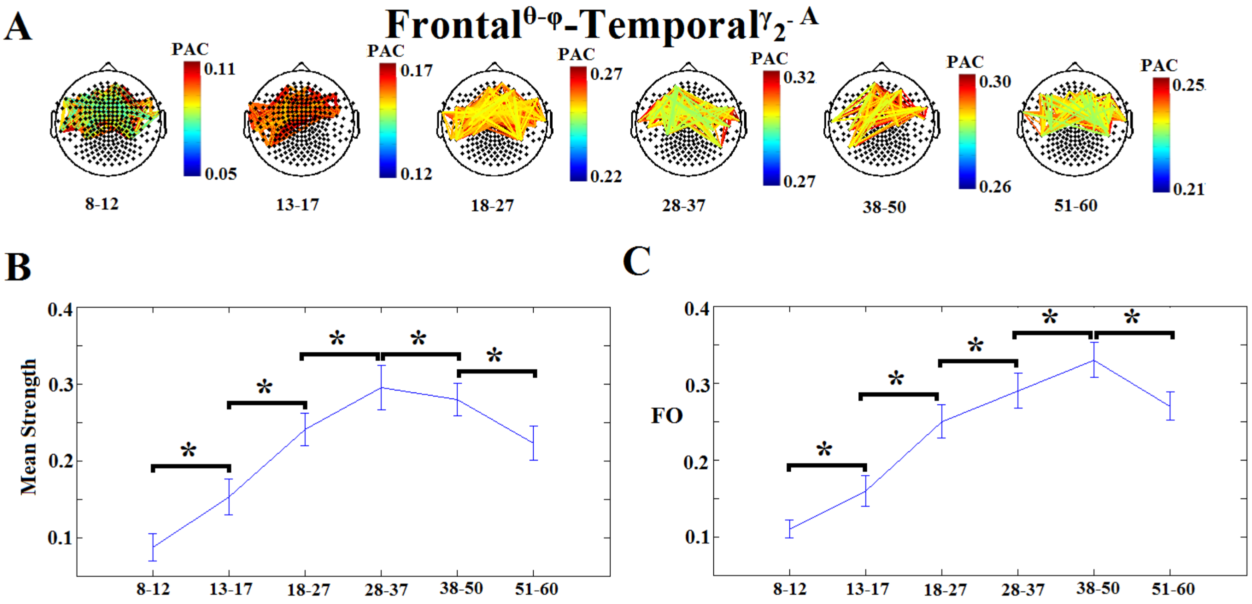

Figure S4. Dominant inter- and intra-hemispheric Phase-Amplitude Coupling (PAC) between frontal and temporal sensors in the θ and γ_2 bands, respectively. A) Topographical layout of statistically significant sensor pairs for the six age groups. B) Mean Subgraph Strength and C) Fractional Occupancy (FO) derived from PAC across the six age groups. Significant differences between successive age groups are marked by brackets ($p < .0001$).

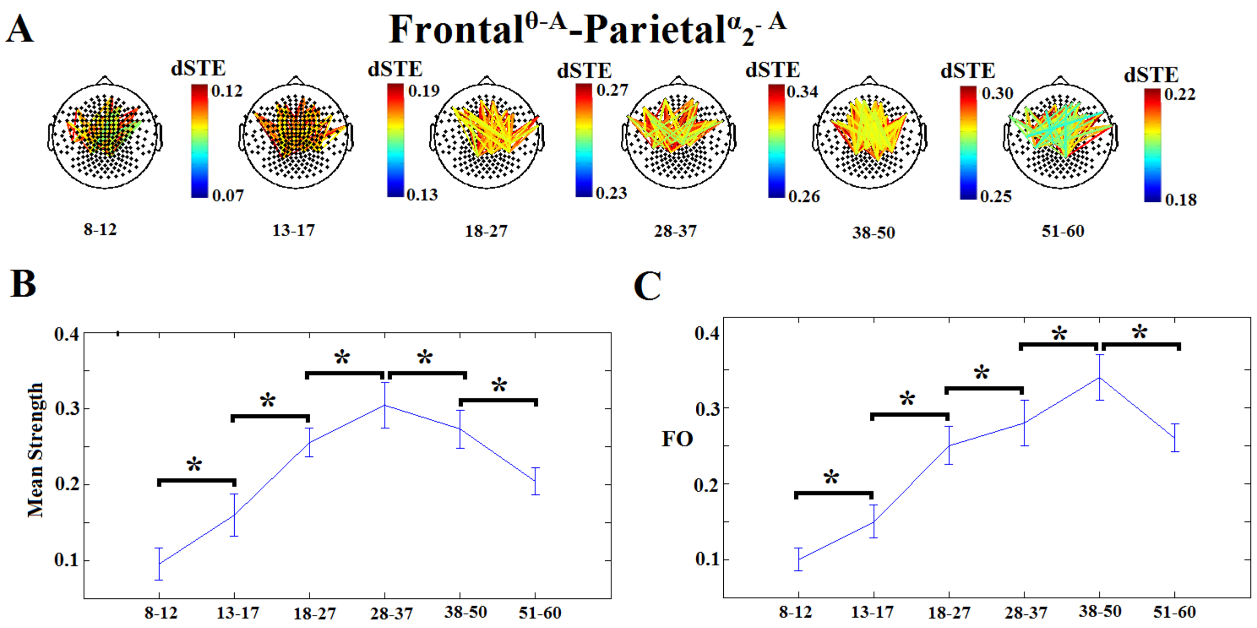

Figure S5. Dominant inter- and intra-hemispheric coupling between frontal and parietal sensors indexed by delay Symbolic Transfer Entropy (dSTE) in the θ and α_2 bands, respectively. A) Topographical layout of statistically significant sensor pairs for the six age groups. B) Mean Subgraph Strength and C) Fractional Occupancy (FO) derived from dSTE across the six age groups. Significant differences between successive age groups are marked by brackets ($p < .0001$).

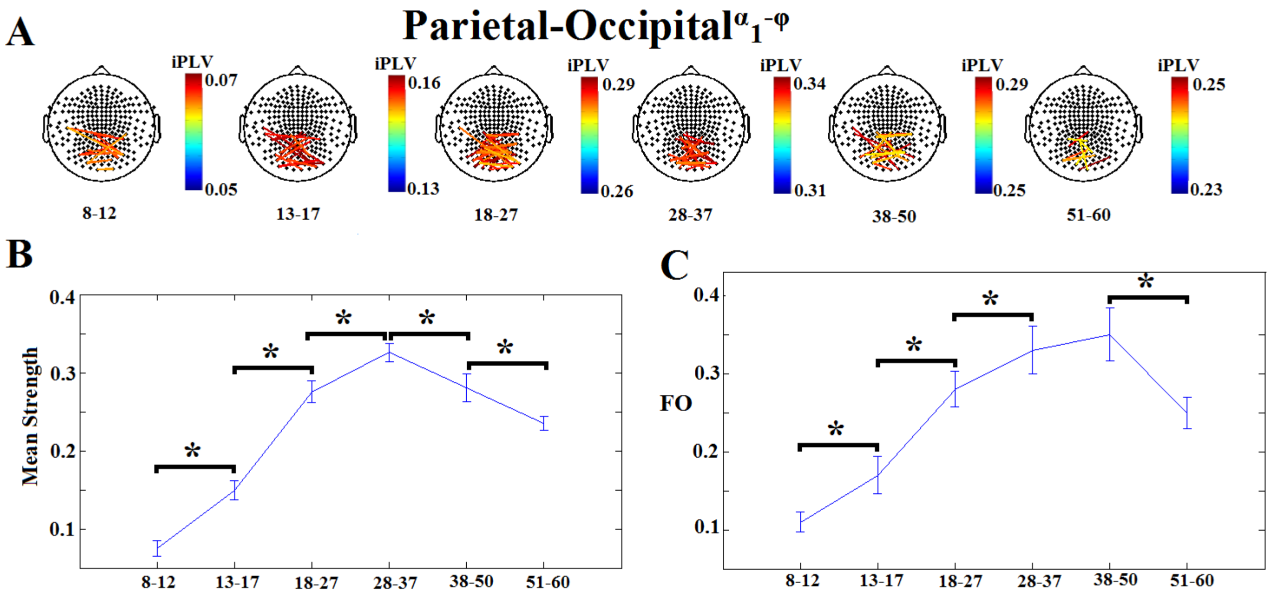

Figure S6. Dominant inter-hemispheric coupling between parietal and occipital sensors indexed by imaginary Phase Locking (iPLV) in the α_1 band. A) Topographical layout of statistically significant sensor pairs for the six age groups. B) Mean Subgraph Strength and C) Fractional Occupancy (FO) derived from iPLV across the six age groups. Significant differences between successive age groups are marked by brackets ($p < .0001$).

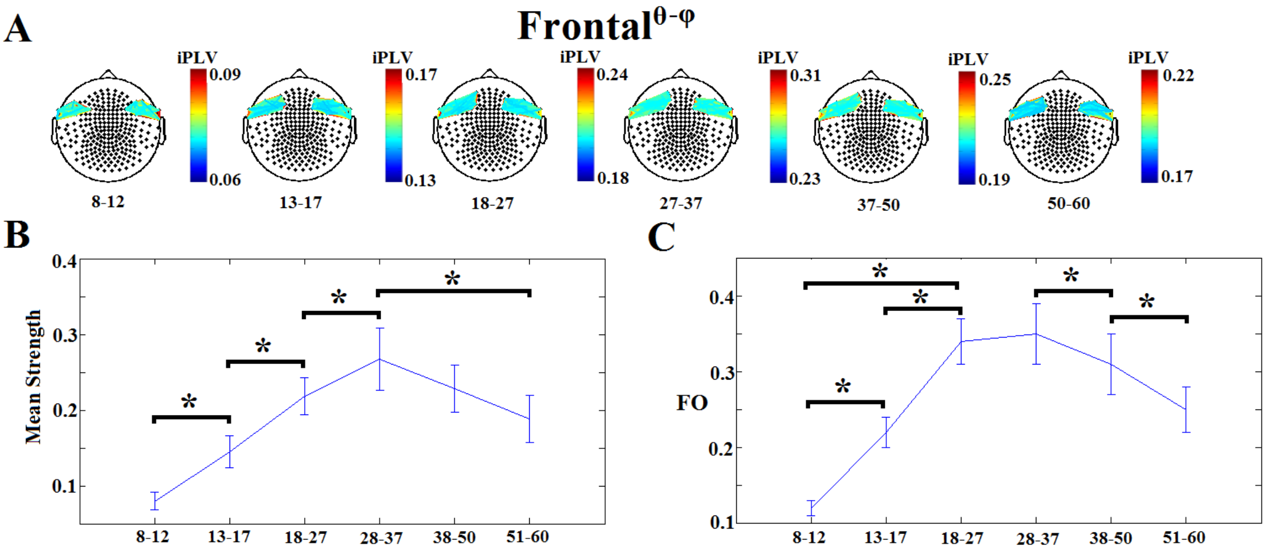

Figure S7. dICM reflecting within-hemisphere phase coupling (imPLV) involving sensors located over the frontal lobes in the θ band. A) Topographic layout of the statistically significant sensor pairs for the six age groups. B) Mean subgraph strength and C) Fractional Occupancy (FO) across the six age groups.

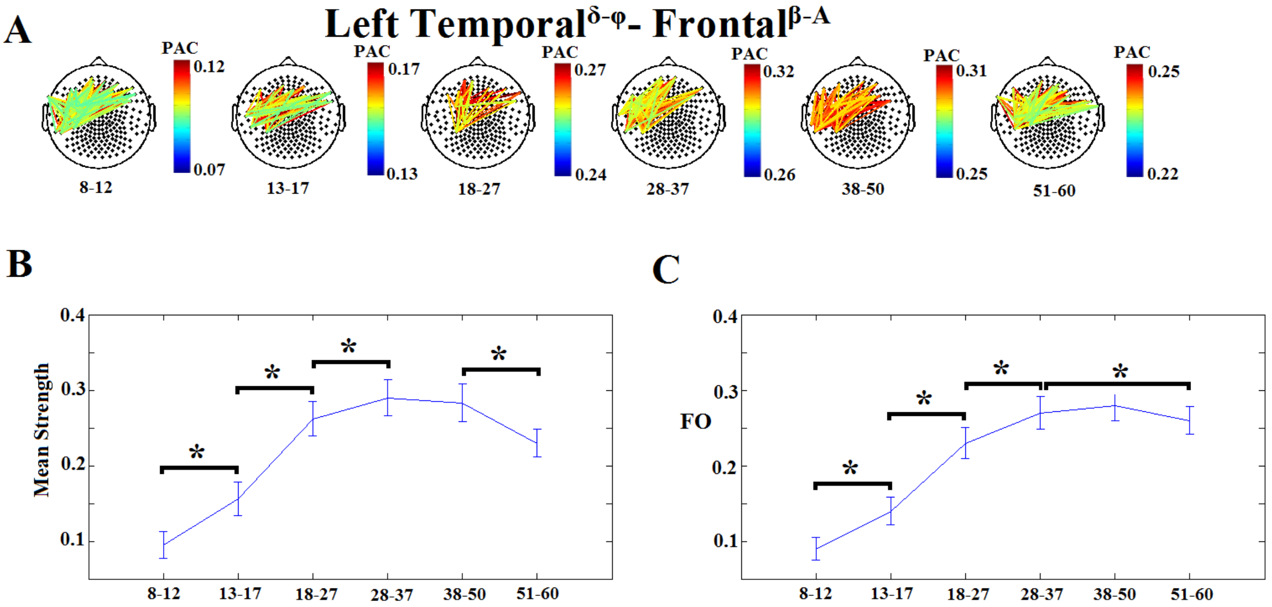

Figure S8. dICM reflecting phase-to-amplitude coupling (PAC) between left temporal sensors in the δ band and bilateral frontal sensors in the β band. A) Topographic layout of the statistically significant sensor pairs for the six age groups. B) Mean subgraph strength and C) Fractional Occupancy (FO) across the six age groups.

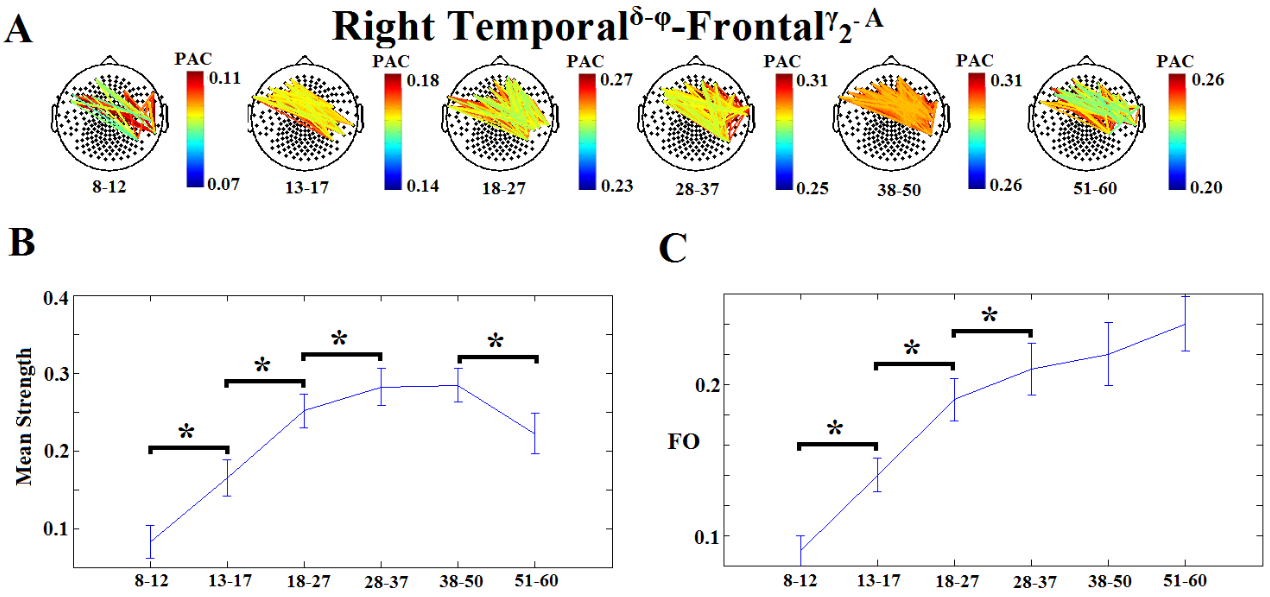

Figure S9. dICM reflecting phase-to-amplitude coupling (PAC) between right temporal sensors in the δ band and bilateral frontal sensors in the γ_2 band. A) Topographic layout of the statistically significant sensor pairs for the six age groups. B) Mean subgraph strength and C) Fractional Occupancy (FO) across the six age groups.

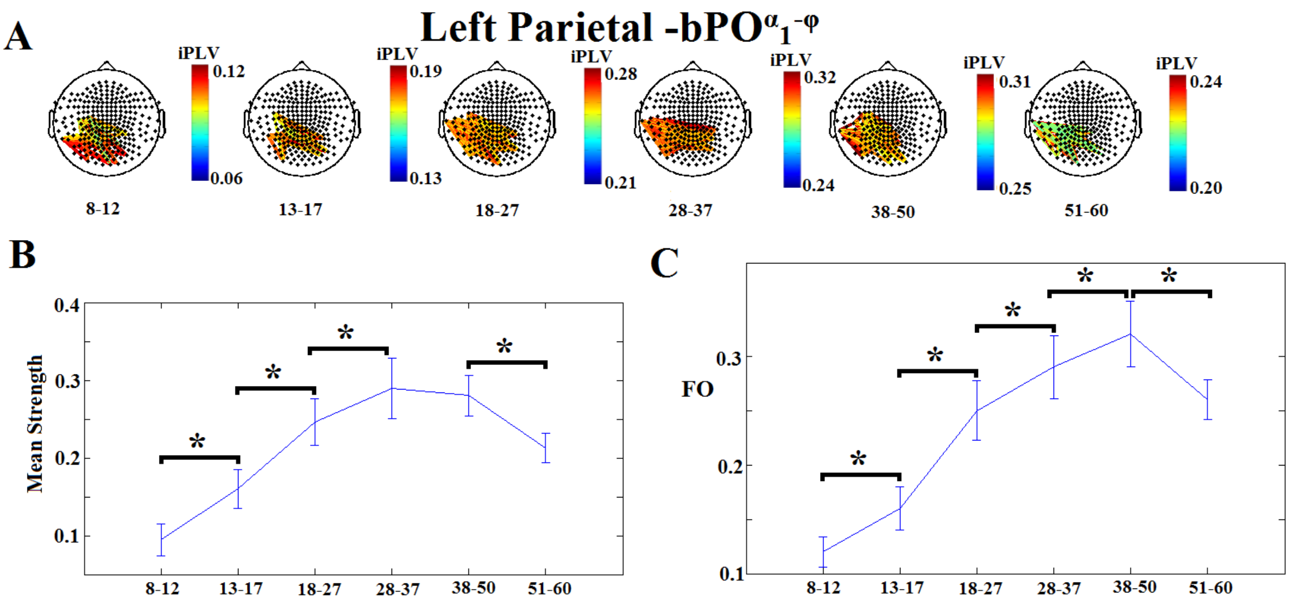

Figure S10. dICM reflecting intra- and inter-hemispheric coupling between bilateral parieto-occipital (bPO) sensors as indexed by the imaginary portion of Phase Locking Value (imPLV) in the α_1 band. A) Topographic layout of the statistically significant sensor pairs for the six age groups. B) Mean subgraph strength and C) Fractional Occupancy (FO) across the six age groups.

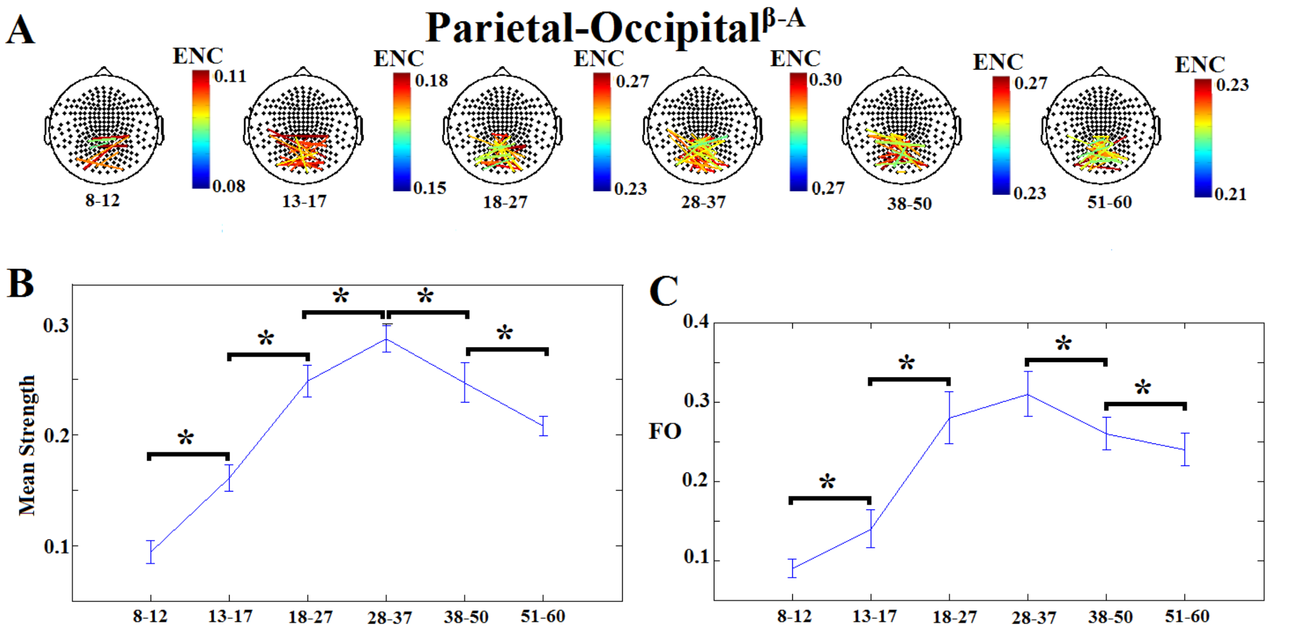

Figure S11. dICM reflecting inter-hemispheric coupling between parieto-occipital sensors as indexed by amplitude envelope correlation (AEC) in the β band. A) Topographic layout of the statistically significant sensor pairs for the six age groups. B) Mean subgraph strength and C) Fractional Occupancy (FO) across the six age groups.

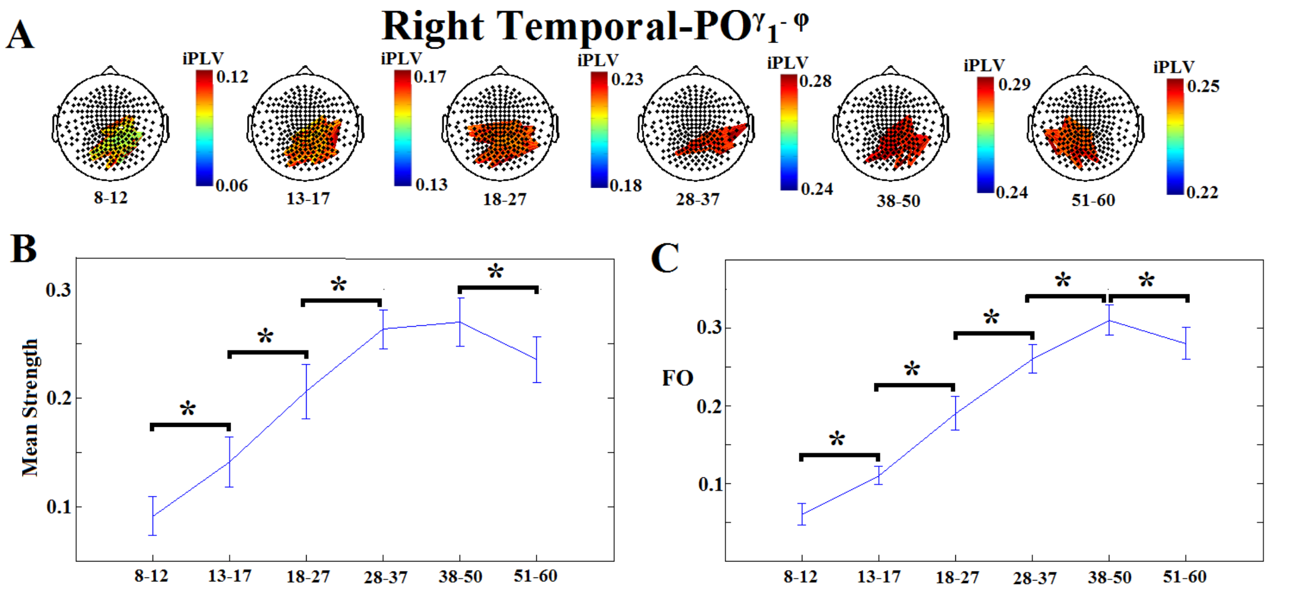

Figure S12. dICM reflecting intra- and inter-hemispheric coupling between right temporal and parieto-occipital sensors (PO) indexed by the imaginary portion of Phase Locking Value (imPLV)in the γ_1 band. A) Topographic layout of the statistically significant sensor pairs for the six age groups. B) Mean subgraph strength and C) Fractional Occupancy (FO) across the six age groups.

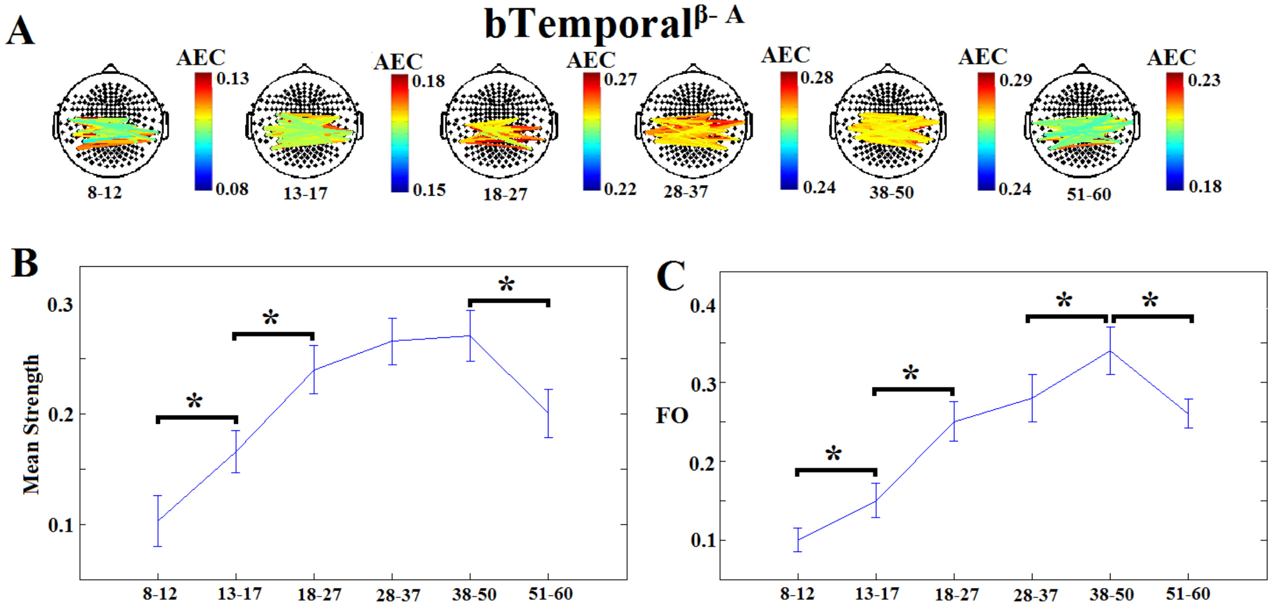

Figure S13. dICM reflecting interhemispheric interactions between temporal sensors indexed by amplitude envelope correlation (AEC) in the β band. A) Topographic layout of the statistically significant sensor pairs for the six age groups. B) Mean subgraph strength and C) Fractional Occupancy (FO) across the six age groups.

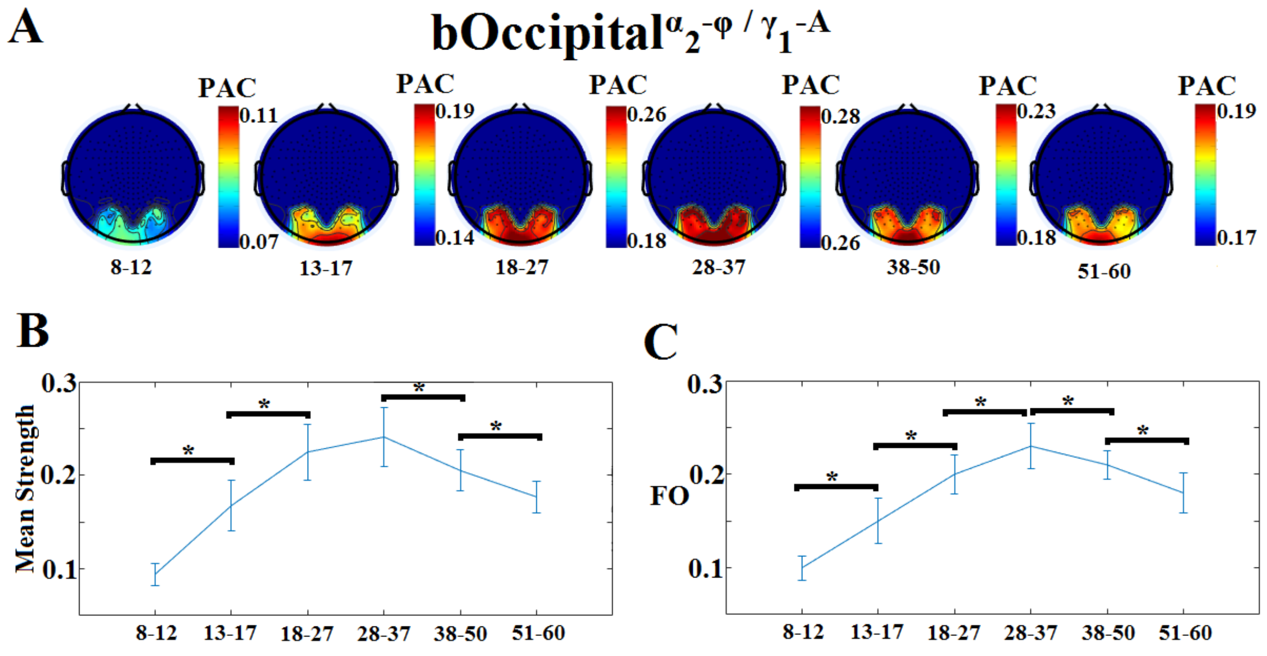

Figure S14. dICM reflecting interhemispheric interactions between occipital sensors indexed by phase-to-amplitude coupling (PAC) in the α_2 and γ_1 bands, respectively. A) Topographic layout of statistically significant sensor pairs for the six age groups. B) Mean subgraph strength and C) Fractional Occupancy (FO) across the six age groups.

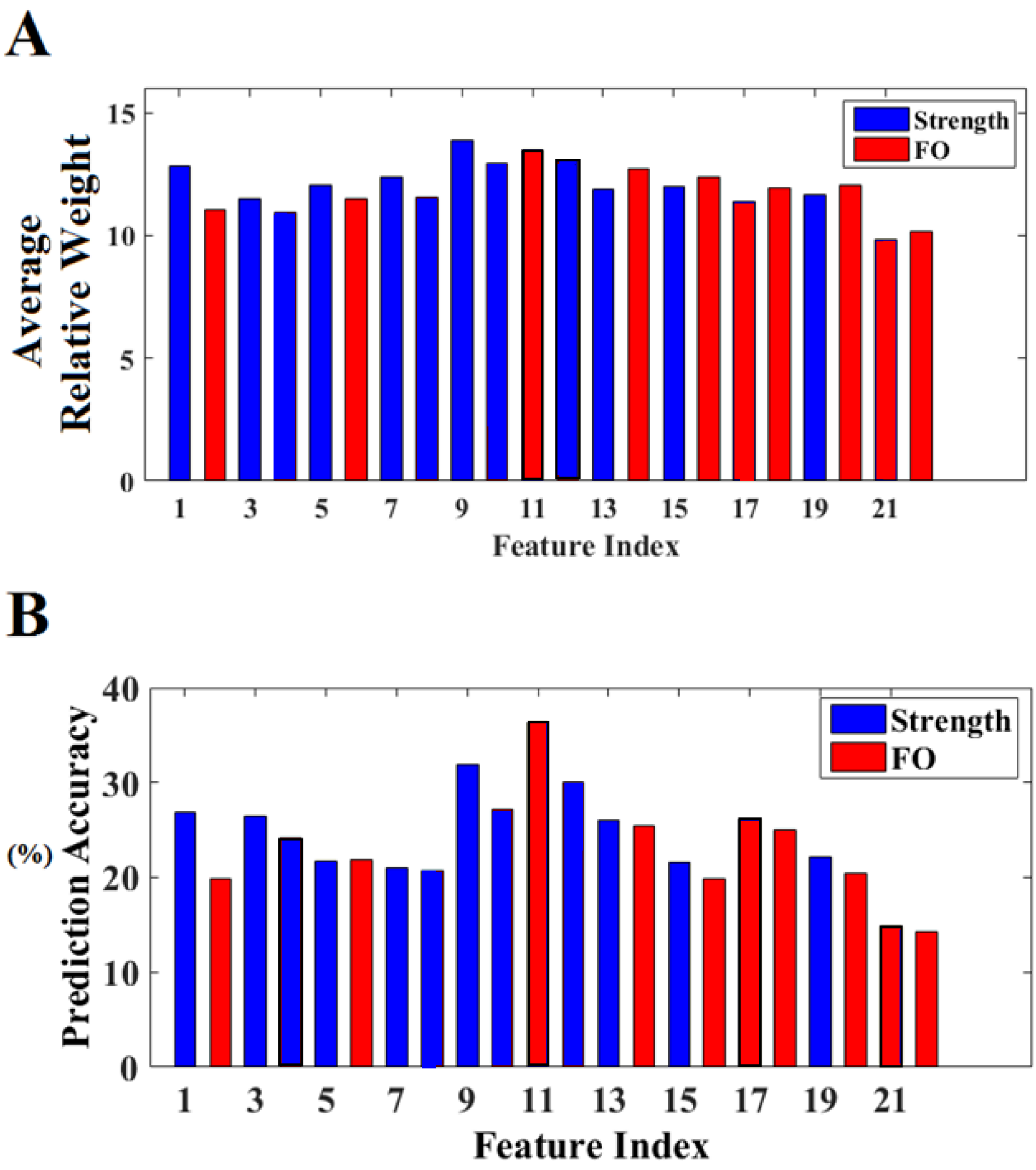

Figure S15. **(A)** Relative weights for Mean Subgraph Strength (MSS; “Strength”) and Fractional Occupancy (FO) for 12 and 10 sub-networks, respectively, representing lobar dynamic connectivity estimates (dICMs) in the prediction of participant age. **(B)** Prediction accuracy of participant age achieved by each of the 22 features.

6.3. *Age-Related Differences based on supplementary indices*

6.3.1. Relative Power

Figure S16 displays average RP values at each frequency band across the six age groups. The prediction of age though a linear combination of 12 RP features was significant, albeit considerably poorer than the prediction using dICM features (R^2=0.430, p < 3.1 x 10-4, see Figure S17). The most consistent age-related features corresponded to data from 12 sensors located over Frontal and Parieto-Occipital regions, the individual contribution of which to the prediction of participant age is shown in Figure S18.

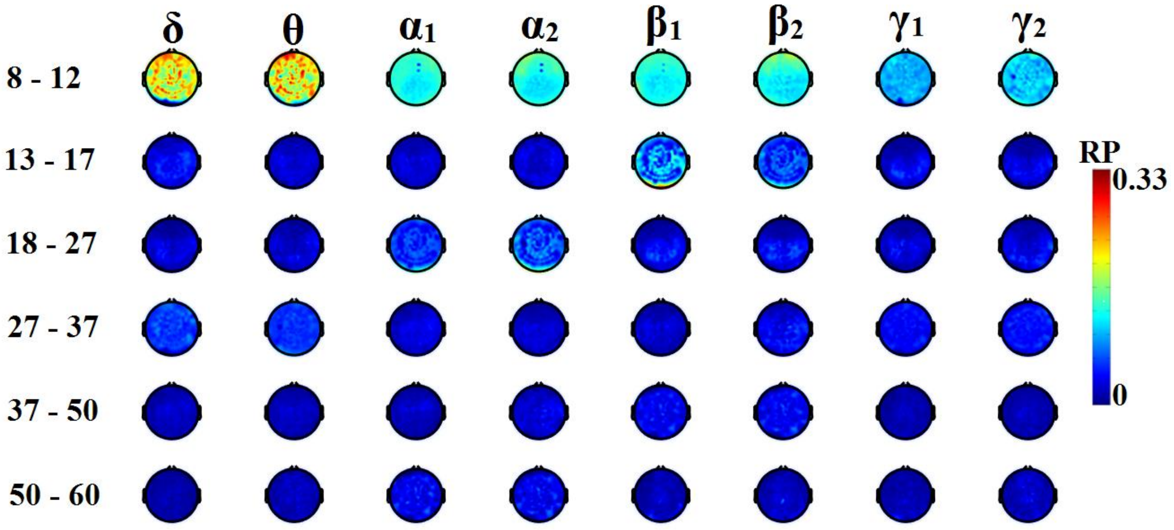

Figure S16. Mean Relative Power (RP) across age groups (rows) and frequency bands (columns).

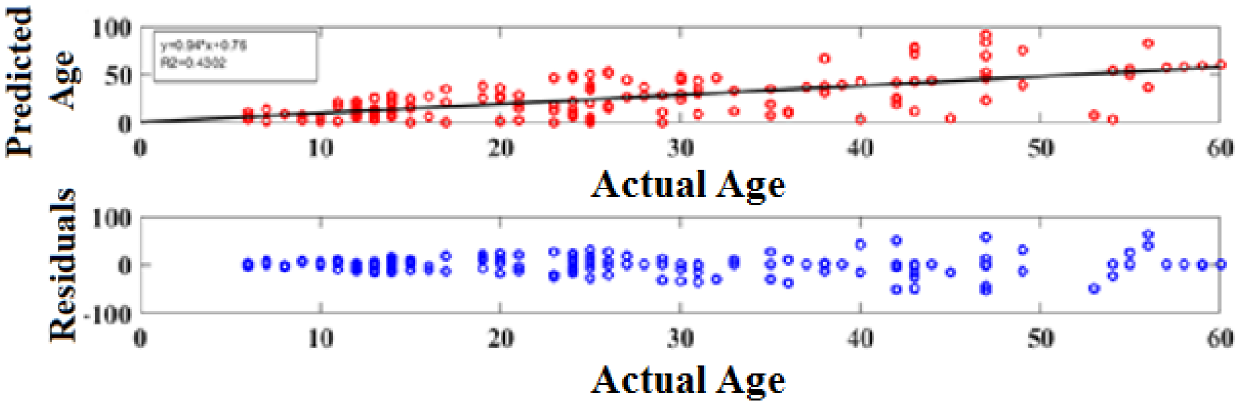

Figure S17. (Upper panel) Least squares regression of predicted over actual participant age (in years) based on Relative Power (RP). (Lower panel) The distribution of model regression residuals as a function of participant age.

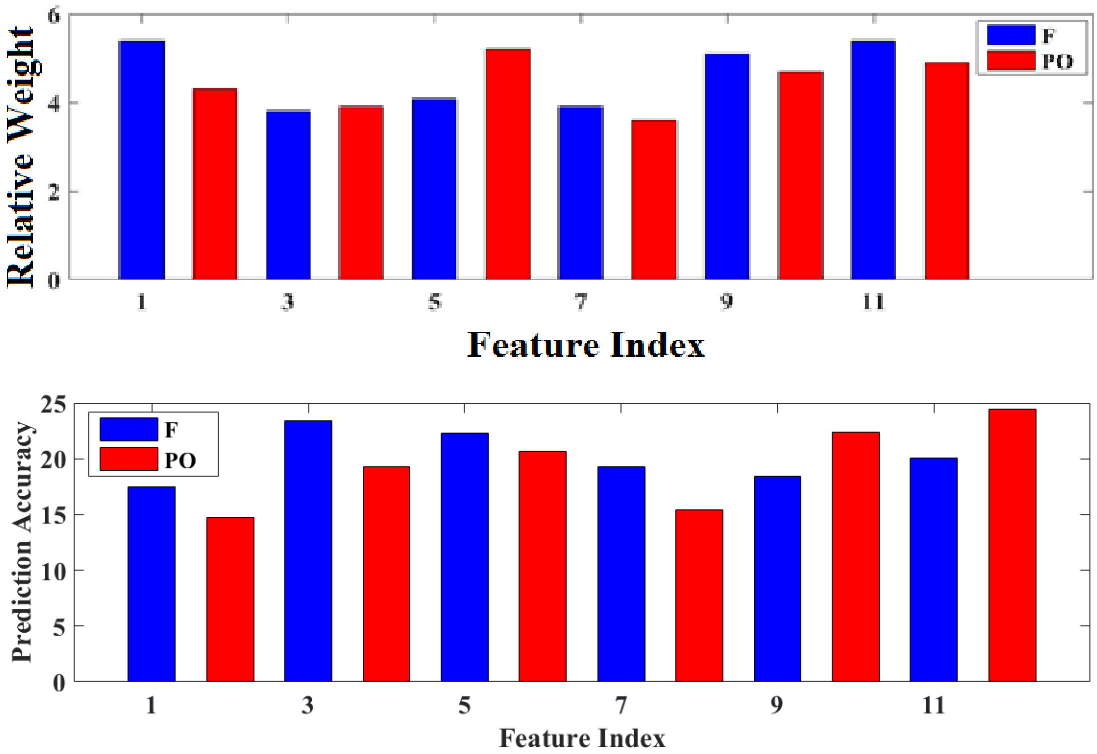

Figure S18. Relative weight (absolute values; upper panel) and individual prediction accuracy (lower panel) of participant age for each of the 12 RP features derived from Frontal (F) and Parieto-Occipital sensors (PO) in the final Support Vector Regression model.

6.3.2. Imaginary Coherence

Figure S19 displays Mean Subgraph Strength values within and between lobar regions in each frequency band. Figure S20 illustrates the significant prediction of age via linear SVR (R^2=0.53, p < 1.9 x 10^{-6}) based on 10 ImCOH features. The relative contribution of individual features significantly contributing to the SVR classification model is shown in Figure S21.

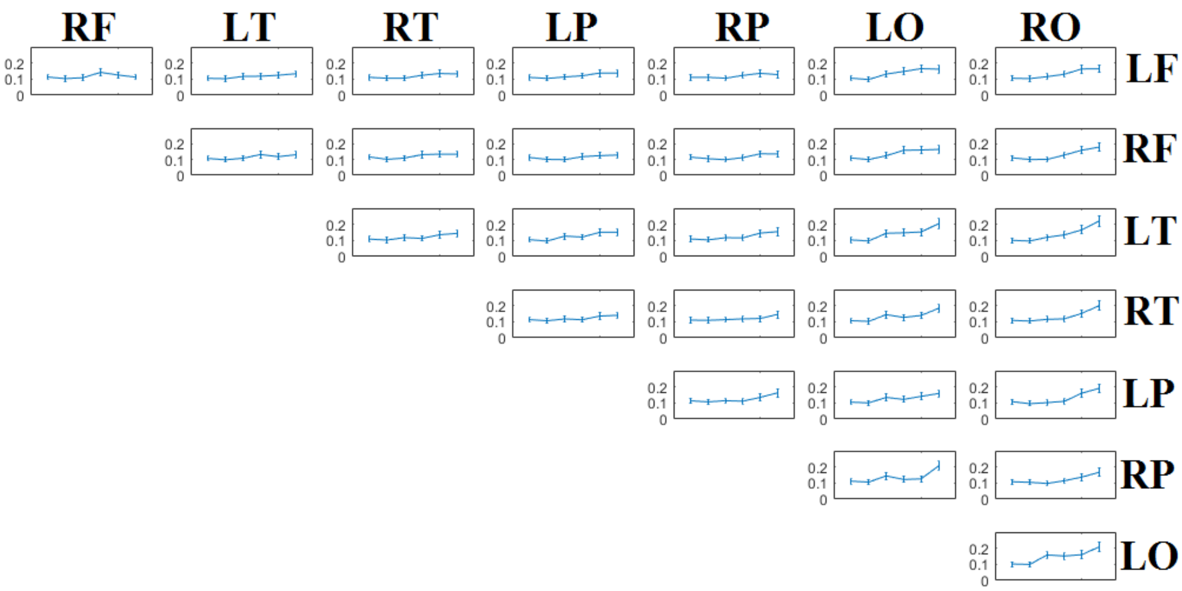

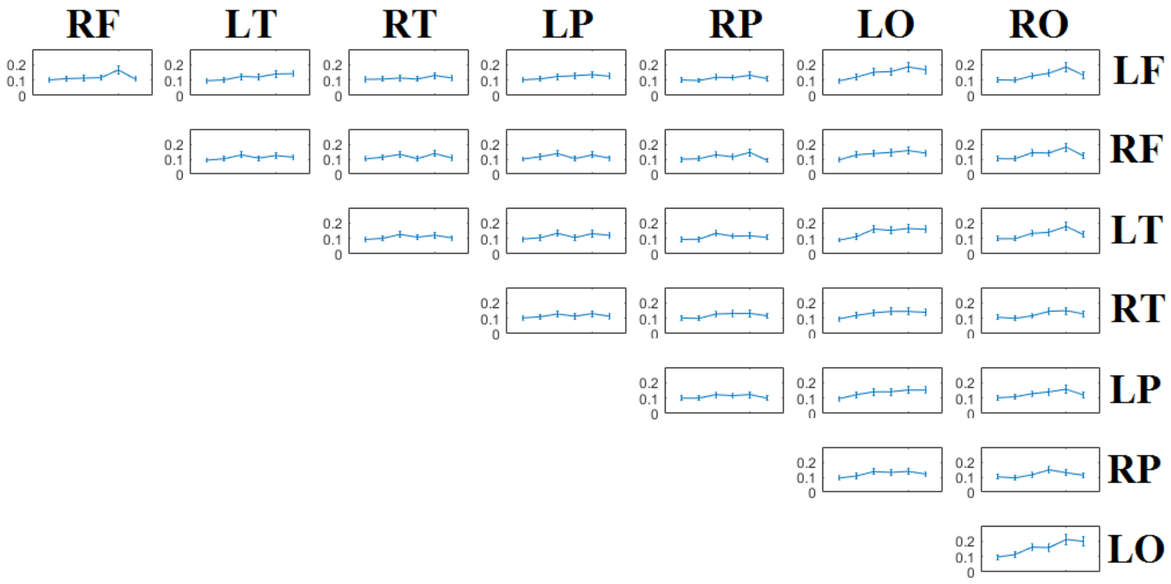
RF LT RT LP RP LO RO
LF RF LT RT LP RP LO

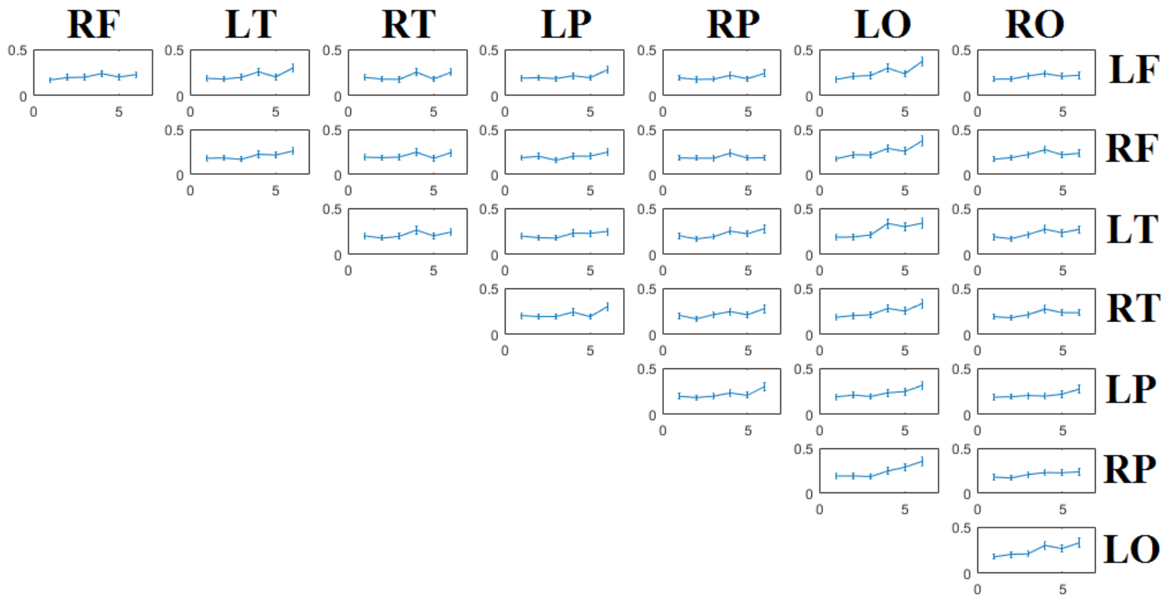
RF LT RT LP RP LO RO
LF RF LT RT LP RP LO

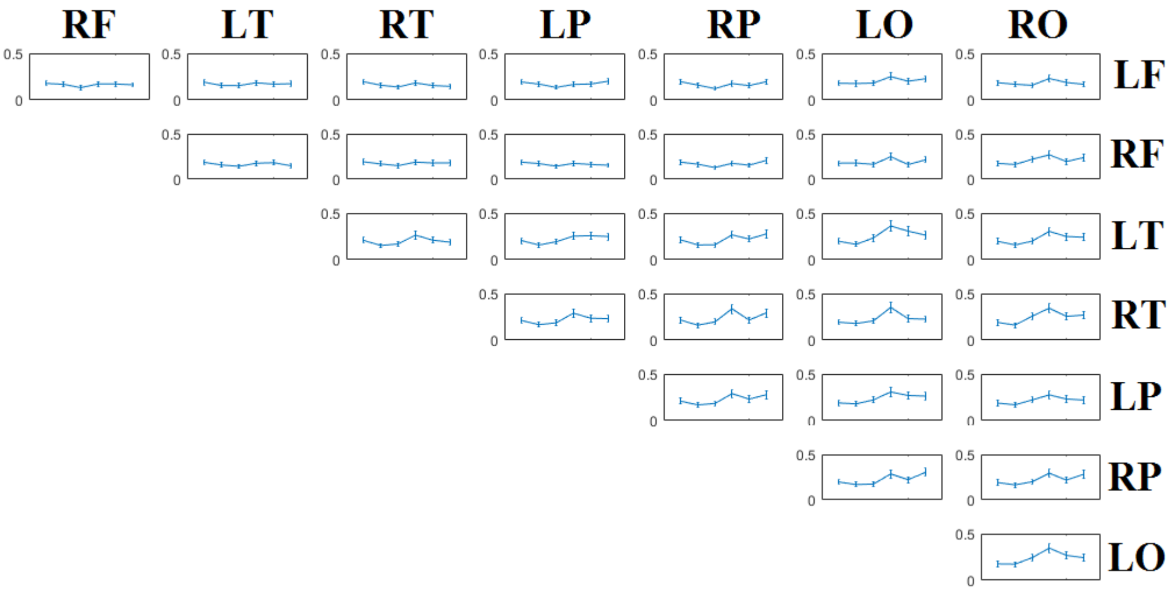
RF LT RT LP RP LO RO
LF RF LT RT LP RP LO

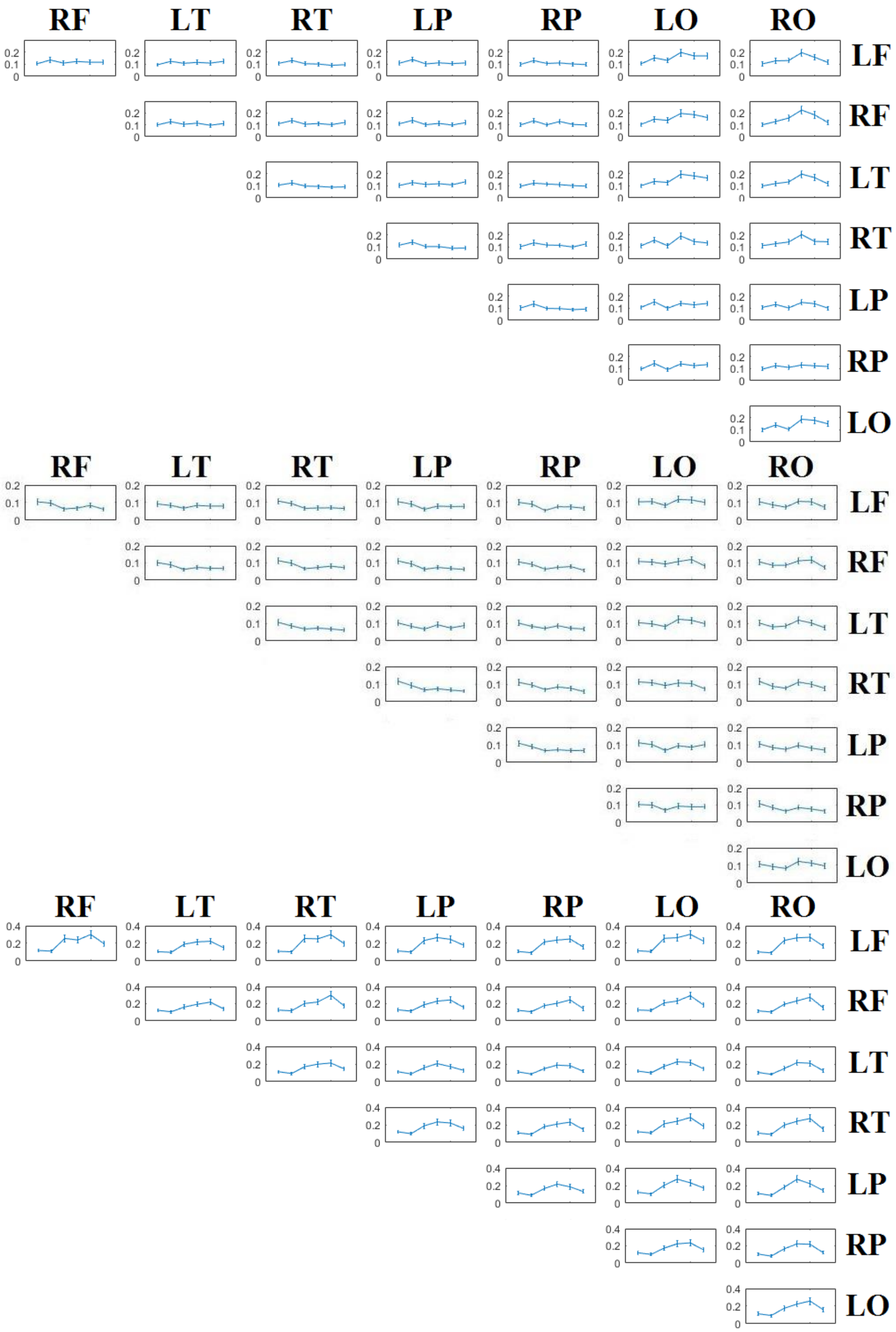

Figure S19. Mean Subgraph Strength reflecting lobar inter- and intra-hemispheric interactions for imCOH across the 6 age groups in eight different frequency bands ranging from δ (top panel) to γ_2 (bottom panel). Abbreviations; L/R: Left/Right hemispheres; F/T/P/O: Frontal/ Temporal/ Parietal/ Occipital lobes.

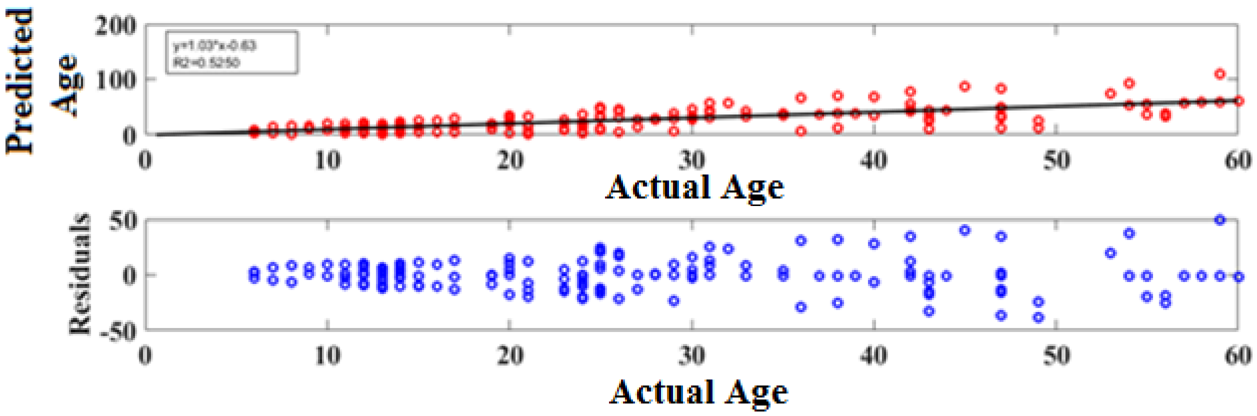

Figure S20. (Upper panel) Least squares regression of predicted over actual participant age in years based on imCOH. (Lower panel) The distribution of model regression residuals as a function of participant age.

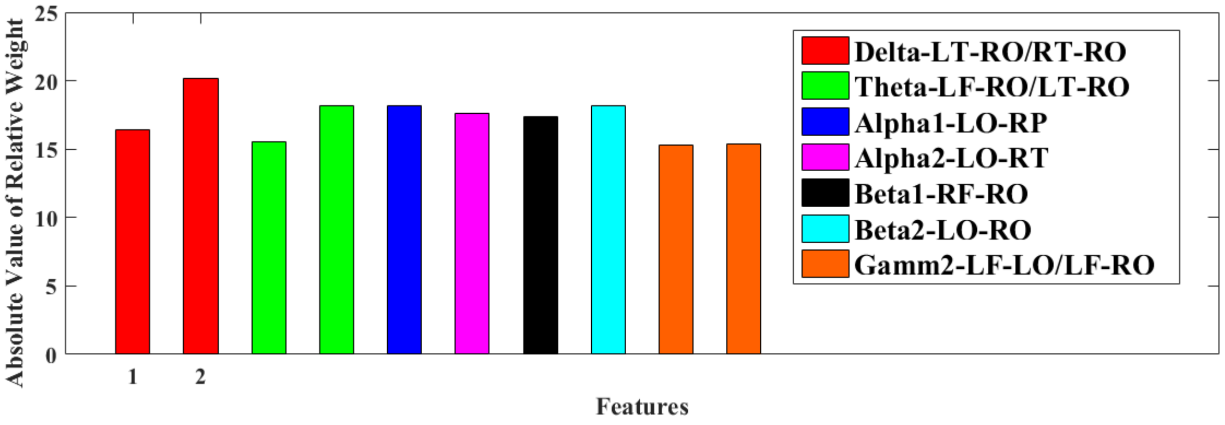

Figure S21. Relative weights for the prediction of participant age for each of the 10 ImCoh features in the final Support Vector Regression model reflecting coherence in various frequency bands between the lobar regions listed in the inset.

6.3.3. Multiscale Entropy

Figure S22 demonstrates the lobe-averaged MSE profile for each age group and frequency band. Figure S23 shows the 4 features that significantly correlated with age and, combined, resulted in the prediction of age via linear SVR (R^2=0.71, $p < 2.9 \times 10^{-6}$) demonstrated in Figure S24. The relative contribution of individual features significantly contributing to the SVR classification model is shown in Figure S25.

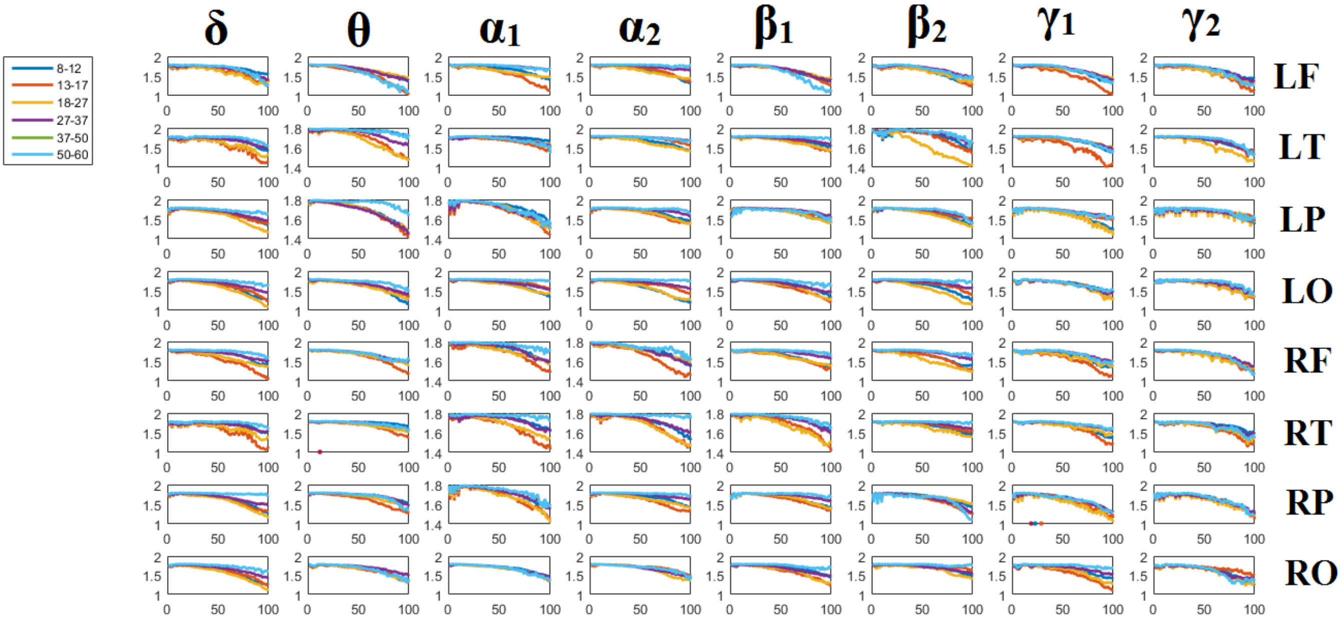

Figure S22. MSE profiles through development. Age-specific MSE profiles across frequency band and lobes (* $p < 0.001$, ANOVA, Bonferroni corrected). Colored lines correspond to one of the six age groups listed in the inset. Abbreviations; L/R: Left/Right hemispheres; F/T/P/O: Frontal/Temporal/Parietal/Occipital lobes.

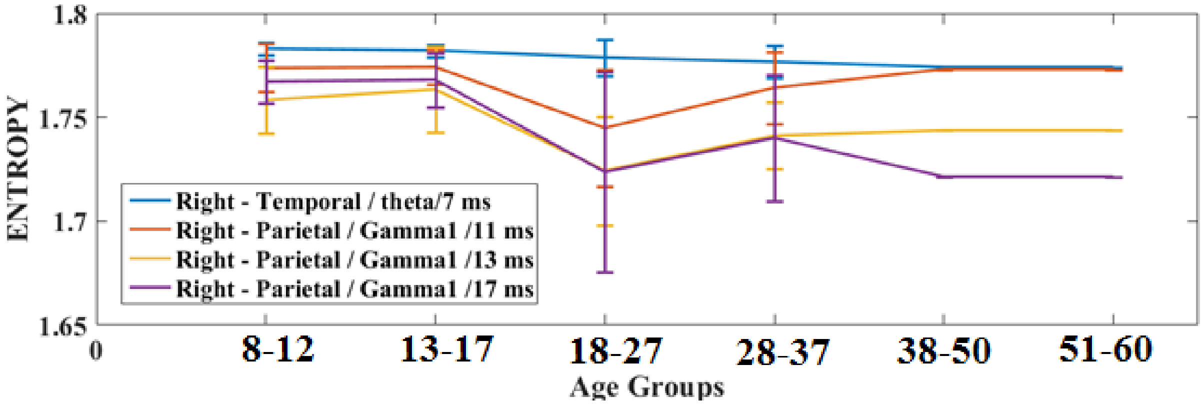

Figure S23. Four features extracted that correlated with age (* $p < 0.001$, ANOVA, Bonferroni corrected; across frequency bands and lobes).

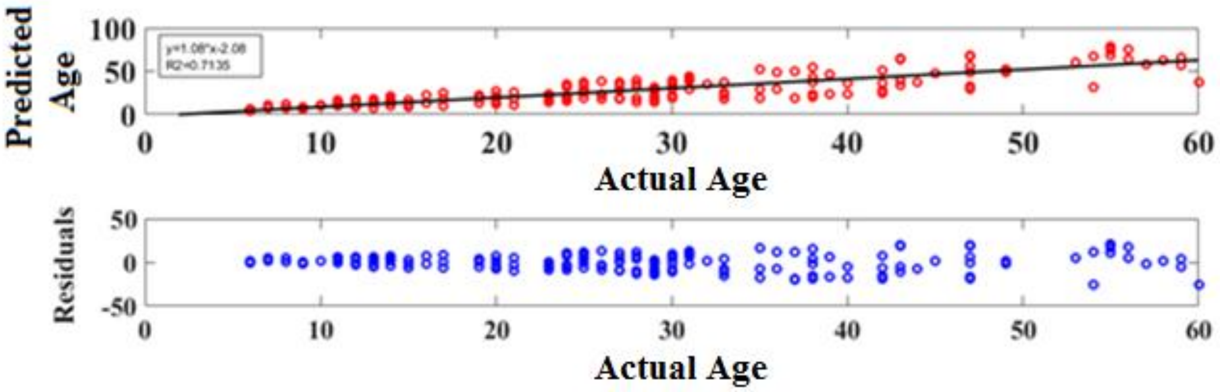

Figure S24. (Upper panel) Least squares regression of predicted over actual participant age in years based on the four MSE features shown in Figure S23. (Lower panel) Distribution of model regression residuals as a function of participant age.

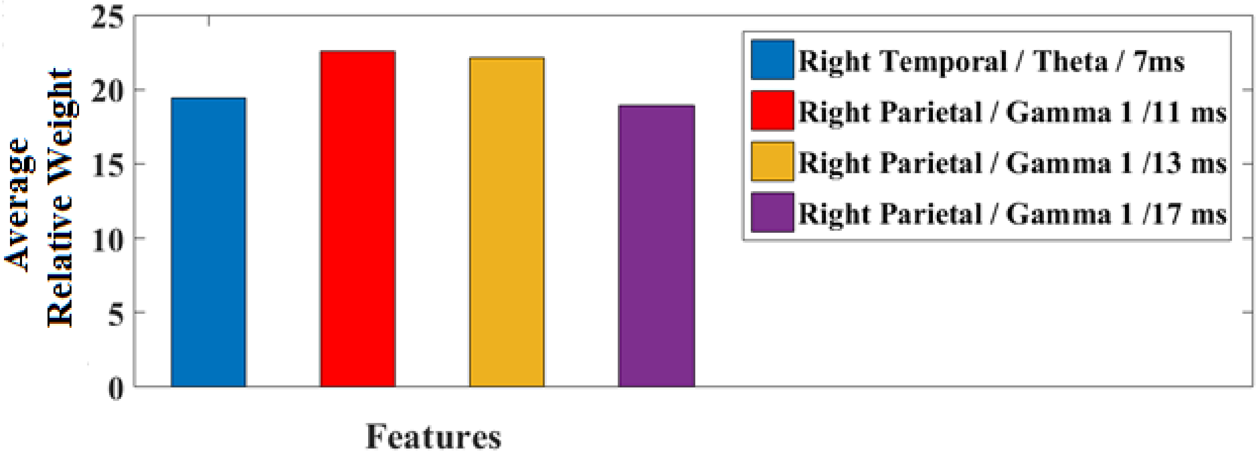

Figure S25. Relative contribution of each of the four MSE features to the linear kernel SVR predictor.

6.3.4. Prediction of participant age using RP, ImCoh and MSE features combined

By comparison the capacity of supplementary metrics (RP, ImCoh, and MSE combined) to predict age via regression analysis demonstrated similar results (R^2=0.812, p < 2.2× 10^{-7}; Fig. S26 & S.Table 1).

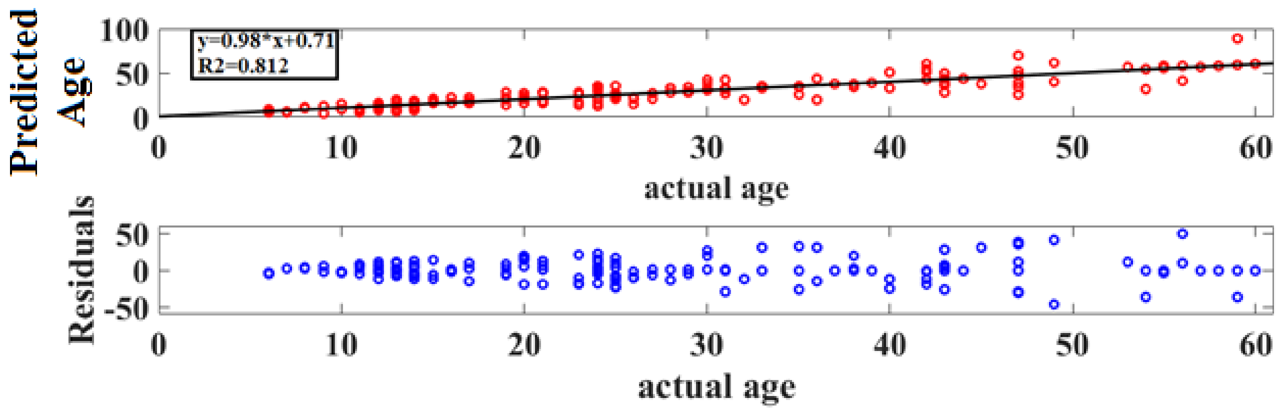

Figure S26. (Upper panel) Least squares regression of predicted over actual participant age in years based on RP, MSE and ImagCoh features combined. (Lower panel) Distribution of model regression residuals as a function of participant age.

6.4. MEG system equivalence

Results from the cross-validation schemes using dICM-based features derived from one MEG system to train the Support Vector Regressor and Multi-Class Support Vector Machine, revealed performance that was comparable, albeit somewhat lower, than the performance obtained when using mixed data sets for training and testing. The prediction of age was similar across systems using the same set of features each time: R^2=0.876, p < 1.8× 10^{-7} when using data from the Magnes-248 system as training-set and R^2=0.869, p < 3.5× $10^{-7}$when using data from the CTF-275 as training set (S.Table 1).

Moreover, classification of participants to the correct age group averaged $86.65 \pm 5.77\%$ when using data from the Magnes-248 system as the training set, and $86.09 \pm 6.03\%$ when using data from the CTF-275 system as the training set (S.Table 2). The similarity of subnetwork topography and age

trends across systems is illustrated in Figure S27 using the dCIM between Frontal lobes as example. Estimates of FI were also comparable across systems as demonstrated by the close similarity of the maturation curves in Figure S28.

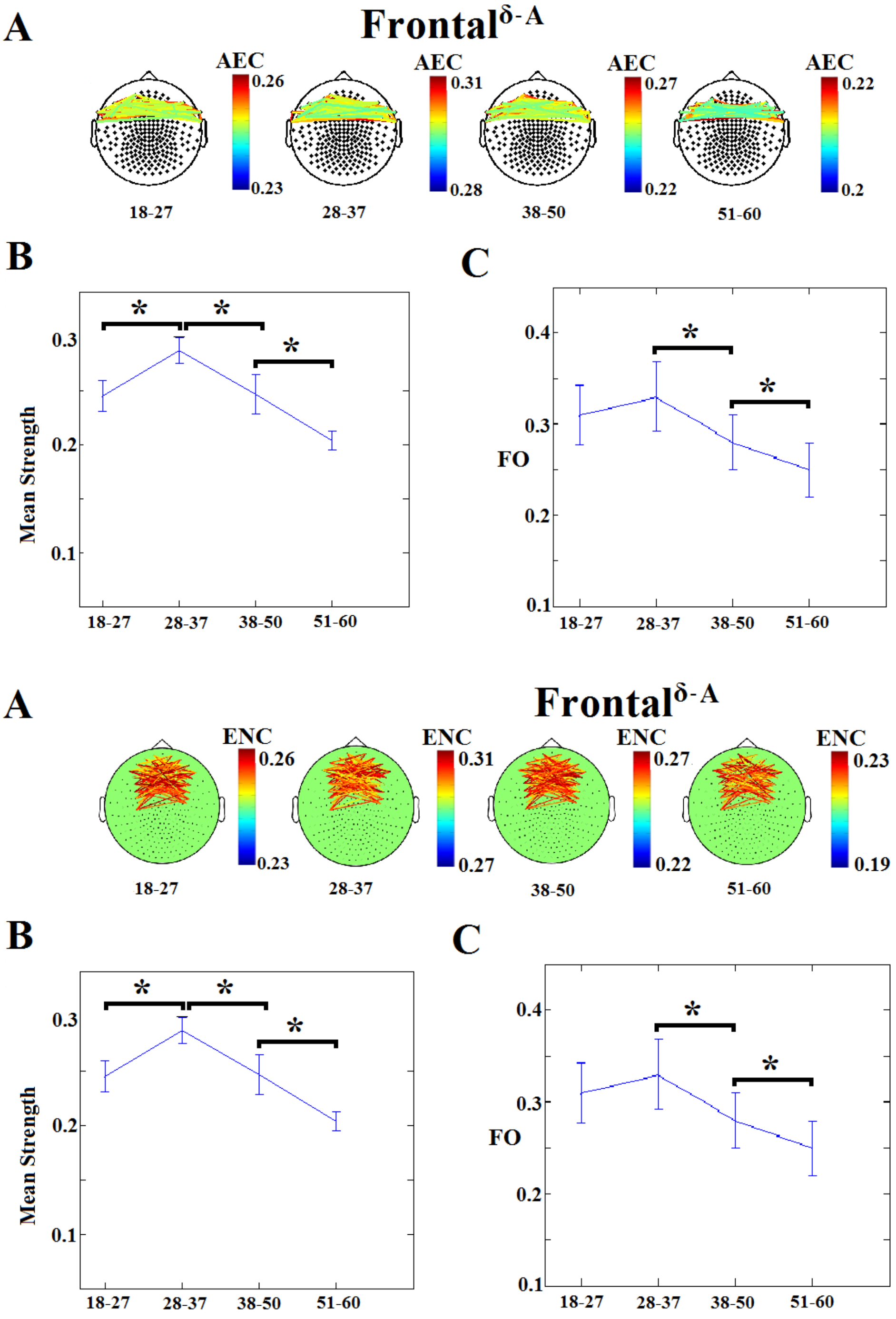

Figure S27. dICM reflecting interhemispheric interactions between frontal sensors as indexed by amplitude envelop correlation in the δ band (AEC). A) Topographical layout of the statistically significant sensor pairs for 4 age groups (overlapping age range between the two MEG systems). B) Mean subgraph strength and C) Fractional Occupancy (FO) across the four age groups. The upper half of the figure presents data recorded on the Magnes-248 system and the lower half displays data recorded on the CTF-275 system.

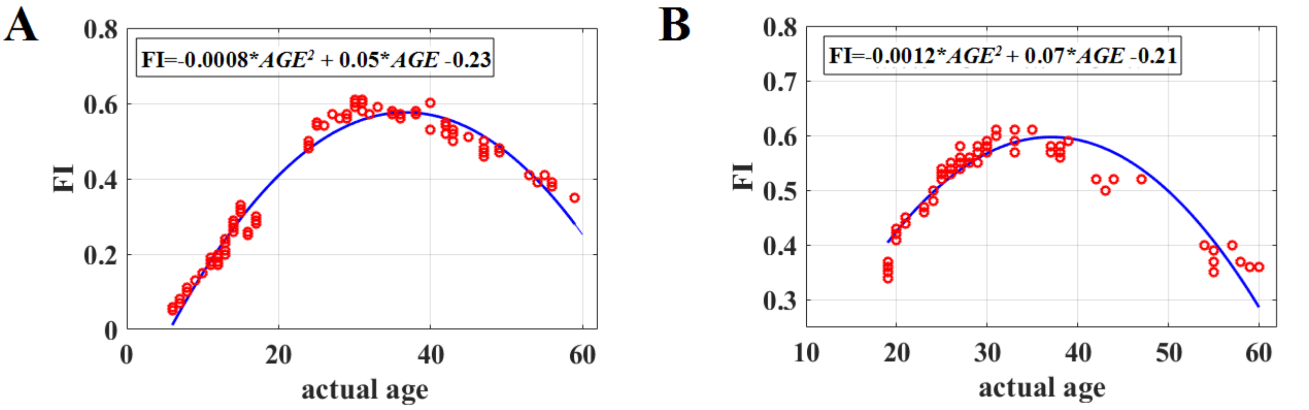

Figure S28. Functional brain maturation curves based on the Flexibility Index (FI) computed for data obtained on the Magnes-248 (A; n=81 aged 6-59 years) and CTF-275 MEG systems (B; n=97 aged 18-60 years). Chronological age is shown on the *x* axis. The best-fitting curves for the data for each system are shown by the blue lines.

6.5. Reliability of the Flexibility Index

One-week test-retest assessment of FI values indicated excellent reliability (r = 0.94) with average test-retest differences = 0.027±0.015, range 0.012-0.029 (see Figure S29).

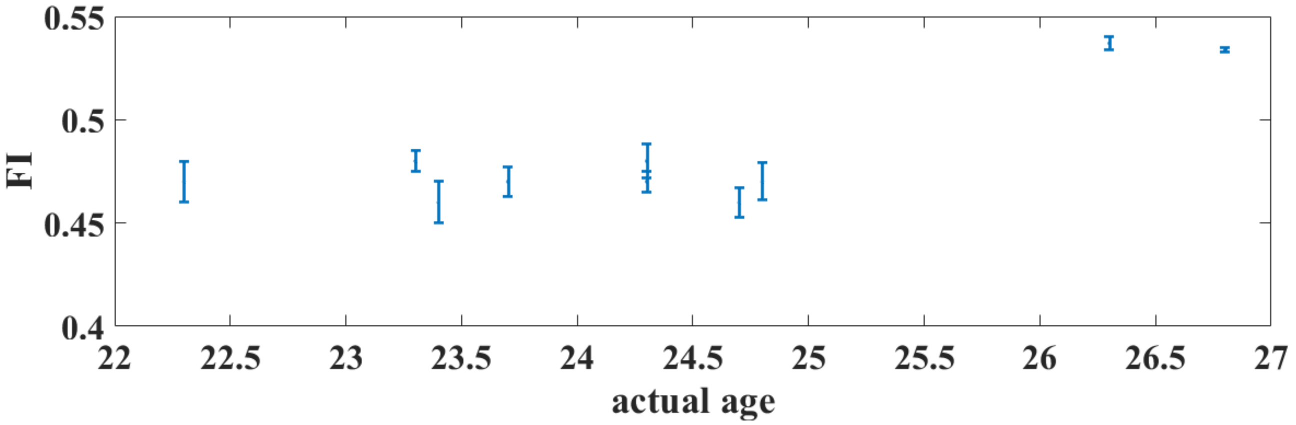

Figure S29. Test-retest Flexibility Index (FI) values as a function of participant age (n=10).

References

Adhikari A, Sigurdsson T, Topiwala MA, Gordon JA. (2010). Cross-correlation of instantaneous amplitudes of field potential oscillations: a straightforward method to estimate the directionality and lag between brain areas. J. Neurosci. Methods, 191, 191-200.

Antonakakis M. et al. (2016). Altered cross-frequency coupling in resting-state MEG after mild traumatic brain injury. Int J Psychophysiol, 102, 1-11.

Aru J, Aru J, Priesemann V, Wibral M, Lana L, Pipa G, Singer W, and Vicente R. (2014) Untangling cross-frequency coupling in neuroscience. arXiv:1405.7965, http://arxiv.org/abs/1405.7965.

Bassett DS, Bullmore ET. (2009). Human brain networks in health and disease. Curr Opin Neurol. 22, 340-7.

Bassett DS, Wymbs NF, Porter MA, Mucha PJ, Carlson JM, Grafton ST. (2011). Dynamic reconfiguration of human brain networks during learning. Proc Natl Acad Sci USA, 108, 7641–

6.
Bassett DS, Wymbs NF, Rombach MP, Porter MA, Mucha PJ, Grafton ST. (2013). Task-based core-periphery organization of human brain dynamics. Plos Comput Biol, 9: e1003171.
Bassett DS, Yang MZ, Wymbs NF, Grafton ST. (2015). Learning-induced autonomy of sensorimotor systems. Nat Neurosci, 18, 744–51.
Benjamini Y, Hochberg Y. (1995): Controlling the False Discovery Rate - a Practical and Powerful Approach to Multiple Testing. J. Royal Stat. Soc. Ser. B-Stat. Methodol. 57, 289-300.
Buzsáki, G and Watson B.O. (2012). Brain rhythms and neural syntax: implications for efficient coding of cognitive content and neuropsychiatric disease. Dialogues Clin. Neurosci. 14, 345–367.
Buzsáki, G., Logothetis, N. and Singer, W. (2013). Scaling brain size, keeping timing: evolutionary preservation of brain rhythms. Neuron, 80, 751-64.
Canolty, RT and Knight RT. (2010). The functional role of cross-frequency coupling, Trends Cogn. Sci., 14, 506-15.
Chavez M, Martinerie J, Le Van Quyen M. (2003): Statistical assessment of nonlinear causality: application to epileptic EEG signals. J. Neurosci. Methods 124, 113-28.
Corominas-Murtra B, Goñi J, Solé RV, Rodríguez-Caso C. (2013). On the origins of hierarchy in complex networks. Proc Natl Acad Sci 110, 13316–21.
Costa M, Goldberger AL, Peng C-K. (2002). Multiscale entropy to distinguish between physiologic and synthetic RR time series. Comp Cardiol, 29, 137-140.
Costa M, Goldberger AL, Peng C-K. (2005). Multiscale entropy analysis of biological signals. Phys Rev E, 71, 021906
Crammer K. and Singer Y. (2001). On the Algorithmic Implementation of Multi-class SVMs, J Machine Learn Res, 2, 265-292.
Delorme A. & Makeig, S. (2004). EEGLAB: an open source toolbox for analysis of single-trial EEG dynamics including independent component analysis. J. Neurosci. Methods, 134, 9–21
Dimitriadis, S.I., Laskaris, N.A., Tsirka, V., Vourkas, M., Micheloyannis, S., and Fotopoulos, S. (2010a). Tracking brain dynamics via time-dependent network analysis. *J Neurosci Methods 193*, 145-155.
Dimitriadis, S. I., Laskaris, N. A., Tsirka, V., Vourkas, M., and Micheloyannis, S. (2010b). What does delta band tell us about cognitive Processes: a mental calculation study? Neurosci. Lett. 483, 11–15.
Dimitriadis, S.I., Laskaris, N.A., Tsirka, V., Vourkas, M. and Micheloyannis, S. (2012a). An EEG study of brain connectivity dynamics at the resting state. Nonlinear Dynamics, Psychol Life Sci., 16, 5-22.
Dimitriadis, S.I., Kanatsouli, K., Laskaris, N.A., Tsirka, V., Vourkas, M., and Micheloyannis, S. (2012b). Surface EEG shows that Functional Segregation via Phase Coupling contributes to the neural Substrate of Mental Calculations. Brain Cogn, 80, 45–52.
Dimitriadis SI, Laskaris, N.A., Simos, P.G., Micheloyannis, S., Fletcher, J.M., Rezaie, R. and Papanicolaou, A.C. (2013) Altered temporal correlations in resting-state connectivity fluctuations in children with reading difficulties detected via MEG. Neuroimage 83, 307–317.
Dimitriadis, S.I., Laskaris, N.A., Bitzidou, M.A., Tarnanas, I. and Tsolaki, M. (2015a). A novel biomarker of amnestic MCI based on dynamic Cross-Frequency Coupling patterns during cognitive brain responses. Front. Neurosci. 9, 350.
Dimitriadis, SI, Zouridakis, G., Rezaie, R., Babajani-Feremi, A., & Papanicolaou, A.C. (2015b) Functional connectivity changes detected with magnetoencephalography after mild traumatic brain injury. NeuroImage: Clinical 9, 519–531.

Dimitriadis S, Sun Y, Laskaris N, Thakor N, and Bezerianos A. (2016a). Revealing cross-frequency causal interactions during a mental arithmetic task through symbolic transfer entropy: a novel vector-quantization approach. IEEE Trans Neural Syst Rehabil Eng.

Dimitriadis SI et al. (2016b), Greater Repertoire and Temporal Variability of Cross-Frequency Coupling (CFC) Modes in Resting-State Neuromagnetic Recordings among Children with Reading Difficulties. Front. Hum. Neurosci, http://dx.doi.org/10.3389/fnhum.2016.00163

Dimitriadis, S. I., Tarnanas, I., Wiederholdg, M., Wiederholdh, B., Tsolaki, M., and Fleische, E. (2016c). Mnemonic strategy training of the elderly at risk for dementia enhances integration of information processing via cross-frequency coupling. *Alzheimer's Dement.* 2, 241–249.

Dimitriadis, S. I., Sun, Y., Thakor, N. V., and Bezerianos, A. (2016d). Causal interactions between frontal$^{\theta}$–parieto-occipital$^{\alpha 2}$ predict performance on a mental arithmetic task. *Front Hum Neurosci.* 10:454.

Dimitriadis SI, Sallis C, Tarnanas I and Linden DE. (2017a). Topological Filtering of Dynamic Functional Brain Networks Unfolds Informative Chronnectomics: A novel data-driven thresholding scheme based on Orthogonal Minimal Spanning Trees (OMSTs). *Front. Neuroinform.* **11**:28.

Dimitriadis, S. I., Antonakakis, M., Simos, P. G., Fletcher, J., and Papanicolaou, A. (2017b). Data-driven topological filtering based on orthogonal minimal spanning trees: application to multi-group MEG resting-state connectivity. Brain Connect. 7, 661–670.

Dosenbach NU, Nardos B, Cohen AL, Fair DA, Power JD, Church JA et al. (2010). Prediction of individual brain maturity using fMRI. *Science* 329, 1358–1361.

Drucker, H., Burges, C.J.C., Kaufman, L., Smola, A., and Vapnik, V.N. (1997). Support vector regression machines. In M. C. Mozer, J. Jordan, and T. Petsche *(Eds), Advances in Neural Information Processing Systems*, Vol. 9, Cambridge, MA: MIT Press, pp. 155–161.

Granger, CW. (1969). Investigating causal relations by econometric models and cross-spectral methods, *Econometrica*, 424-438.

Grassberger P. (1991). Information and complexity measures in dynamical systems, in Atmanspacher H, and Scheingraber H (eds.), Information Dynamics. New York: Plenum Press, pp. 15-33.

Hastie, T., Tibshirani, R., and Friedman, J. H. (2001). *The Elements of Statistical Learning: Data Mining, Inference, and Prediction*. New York, NY: Springer Publishing Company, Inc.

Heisz JJ, Gould M, McIntosh AR. (2015). Age-related shift in neural complexity related to task performance and physical activity. J Cogn Neurosci, 27, 605-13.

Ito, S., Hansen, M.E., Heiland R. *et al.* (2011). Extending transfer entropy improves identification of effective connectivity in a spiking cortical network model. *PLoS One,* 6, e27431.

Lachaux JP, Rodriguez E, Van Quyen ML, Lutz A, Martinerie J, Varela FJ. (2000). Studying single-trials of phase synchronous activity in the brain. Intern. J. Bifurc. Chaos 10, 2429-2439.

Lake D.E., Richman J.S., Griffin M.P., Moorman J.R. (2002). Sample entropy analysis of neonatal heart rate variability. Am J Physiol Regul Integr Comp Physiol, 283, R789-97.

Lindner, M., Vicente, R., Priesemann V. *et al.* (2011). TRENTOOL: a Matlab open source toolbox to analyze information flow in time series data with transfer entropy. *BMC Neurosci.*, 12, 119, 2011.

Lizier, J. T., Heinzle, J., Horstmann A. *et al.* (2011). Multivariate information-theoretic measures reveal directed information structure and task relevant changes in fMRI connectivity. *J. Comput. Neurosci.*, 30, 85-107.

McIntosh, R., Vakorin, V., Kovacevic, N., Wang, H., Diaconescuand, A., & Protzne, A. B. (2014). Spatiotemporal Dependency of Age-Related Changes in Brain Signal Variability. *Cereb Cortex, 24,* 1806–1817.

Martinetz TM, Berkovich SG, Schulten KJ. (1993): Neural-Gas Network for Vector Quantization and Its Application to Time-Series Prediction. IEEE Trans. Neural Networ. 4, 558-569.

Mengistu H, Huizinga J, Mouret J-B, Clune J. (2016). The Evolutionary Origins of Hierarchy. PLoS Comput Biol 12, e1004829.

Niso G, Rogers C, Moreau JT, Chen LY, Madjar C, Das S, Bock E, Tadel F, Evans AC, Jolicoeur P, Baillet S. (2016). OMEGA: The Open MEG Archive. *Neuroimage, 124*, 1182-7.

Nolte G, Bai O, Wheaton L, Mari Z, Vorbach S, and Hallett M. (2004). Identifying true brain interaction from EEG data using the imaginary part of coherency. *Clin Neurophysiol., 115,* 2292–2307.

Pincus S.M. (1991). Approximate entropy as a measure of system complexity. *Proc Natl Acad Sci USA, 88,* 2297-2301.

Pincus S.M. (2002). Assessing serial irregularity and its implications for health. *Ann N Y Acad Sci, 954,* 245-67.

Ragwitz, M. and Kantz, H. (2002). Markov models from data by simple nonlinear time series predictors in delay embedding spaces. *Phys. Rev. E*, 65, 056201.

Richman J.S., Moorman J.R. (2000). Physiological time-series analysis using approximate entropy and sample entropy. *Am J Physiol Heart Circ Physiol, 278,* H2039-H2049.

Tallon-Baudry C, Bertrand O, Delpuech C, Pernier J. (1997): Oscillatory gamma-band (30-70 Hz) activity induced by a visual search task in humans. *J. Neurosci. 17*, 722-734.

Schreiber T. (2000). Measuring information transfer. Phys. Rev. Lett., 85(2), 461-464.

Shannon, C. E., & Weaver, W. (1949). The Mathematical Theory of Communication. Urbana, IL The University of Illinois Press, 1-117.

Stam, CJ and van Straaten, ECW. (2012). Go with the flow: Use of a directed phase lag index (dPLI) to characterize patterns of phase relations in a large-scale model of brain dynamics. Neuroimage 62, 1415-1428.

Stam CJ. (2014). Modern network science of neurological disorders. *Nat Rev Neurosci* 15, 683–695.

Torrence C, Compo GP. (1998). A practical guide to wavelet analysis. *Bull. Amer. Meteorol. Soc. 79,* 61-78.

Verdes PF. (2005). Assessing causality from multivariate time series. Phys. Rev. E 72, 026222.

Weston, J., Elisseeff, A., Bakir, G., and Sinz, F. (2005). *The Spider Machine Learning Toolbox*. Resource object oriented environment. Available at: http://people.kyb.tuebingen.mpg.de/spider/main.html.

Wibral, M., Pampu, N., Priesemann *V. et al.* (2013). Measuring information-transfer delays. *PLoS One,* 8, e55809.

Zhang J, Cheng W, Liu Z, Zhang K, Lei X, Yao Y, Becker B, Liu Y, Kendrick KM, Lu G, Feng J. (2016). Neural, electrophysiological and anatomical basis of brain-network variability and its characteristic changes in mental disorders. *Brain, 139,* 2307-21.
